# *Porphromonas gingivalis* infection induces gingipain-dependent changes in the brain vasculature of zebrafish larvae

**DOI:** 10.1186/s12964-025-02557-6

**Published:** 2025-11-27

**Authors:** Anna Mieszkowska, Magdalena Marcinkowska, Magdalena Widziolek, Jan Potempa, Magdalena Chadzinska

**Affiliations:** 1 Institute of Zoology and Biomedical Research, Jagiellonian University, Kraków, Poland; 2 Doctoral School of Exact and Natural Sciences, Jagiellonian University, Kraków, Poland; 3 Department of Microbiology, Faculty of Biochemistry, Biophysics and Biotechnology, Jagiellonian University, Krakow, Poland; 4 Department of Oral Immunity and Infectious Diseases, University of Louisville School of Dentistry, Louisville, KY USA

**Keywords:** Brain-blood barrier permeability, Tight junction proteins, *Porphyromonas gingivalis*, Gingipains, Zebrafish larvae

## Abstract

Recent studies link infections with blood–brain barrier (BBB) dysfunctions, neuroinflammation and subsequent neurodegeneration. Here, we employed a zebrafish larval model to study the impact of systemic infection with an oral pathogen *Porphyromonas gingivalis* (*Pg*) and its major virulence factors – gingipains, on BBB integrity and cerebral vasculature. We demonstrated that systemic infection with wild-type *Pg* W83 significantly increased BBB permeability in zebrafish larvae, as evidenced by the extravasation of tracers into the brain parenchyma. This effect was absent in larvae infected with a gingipain-deficient mutant bacteria (ΔK/R-ab), indicating a pivotal role for gingipains in BBB disruption. Immunohistochemical analysis revealed a marked reduction in the expression of tight junction (TJ) proteins: Claudin-5 and Zo-1 on the cerebral vessels of *Pg* W83-infected larvae, while expression of genes encoding TJ proteins (*cldn5a/b* and *tjp1a/b*) was not changed, suggesting post-translational degradation as the primary mechanism. To verify the individual contributions of gingipains, larvae were injected with purified arginine- (RgpA and RgpB) or lysine- (Kgp) specific proteases. In larvae treated with RgpB and Kgp, but not those treated with RgpA, there was increased BBB permeability, loss of TJ proteins, and cerebrovascular changes. Among these, Kgp exerted the most pronounced effects, emphasizing its dominant role in *Pg*-mediated vascular destruction. Our findings for the first time provide *in vivo* evidence that *Pg* compromises BBB integrity via gingipain-dependent degradation of tight junction proteins on the cerebral vessels. Zebrafish larvae offer a robust model for studying *Pg*–BBB interactions and may support the development of therapeutic strategies targeting gingipains to preserve cerebrovascular integrity in neurodegenerative disease.

**Supplementary Information:**

The online version contains supplementary material available at https://doi.org/10.1186/s12964-025-02557-6.

## Introduction

Neurodegenerative disorders are among the prominent causes of disability and death in the aging population. Alzheimer’s disease (AD), the most common form of dementia, is a progressive neurodegenerative condition associated with loss of cognitive and behavioral functions [1, 2]. Despite extensive research, the exact etiology of AD remains not fully understood [3]. While the accumulation of amyloid-β (Aβ) plaques and tau neurofibrillary tangles (NFTs) has long been considered central to AD pathology, increasing evidence suggests that disruption of the blood–brain barrier (BBB) may also play a pivotal role in disease progression [2, 4–6].

The BBB is a highly selective anatomical and functional barrier that separates the central nervous system (CNS) from the systemic circulation, protecting the brain from potentially harmful substances and invading pathogens [7]. It is composed of several interacting cell types, including endothelial cells, pericytes, and astrocyte end-feet [8]. In most vertebrates, endothelial cells lining the cerebral vasculature are sealed by tight junctions (TJs), which are essential for maintaining BBB integrity and regulating molecular permeability [9]. TJs are composed of transmembrane proteins such as claudins and occludins, supported by cytoplasmic scaffold proteins of the zonula occludens (Zo) family, which link junctional complexes to the actin cytoskeleton [10]. Disruption of TJ proteins has been associated with compromised BBB integrity in neurodegenerative disorders, including AD [11].

Recently, a growing body of evidence suggests a link between microbial and viral infections and AD neuropathology. Bacterial DNA, including that of *Chlamydia pneumoniae* and *Borrelia burgdorferi*, has been detected in the CNS of AD patients [12], leading to the hypothesis that AD progression may involve the dissemination of infectious agents within the CNS. Among them, *Porphyromonas gingivalis* (*Pg*), a keystone pathogen in chronic periodontitis (CP), has been implicated in AD due to the presence of its DNA and virulence factors in postmortem brain tissue of AD patients [13]. *Pg* can enter the bloodstream during routine oral activities or dental procedures [14], enabling systemic dissemination.

*Pg* is a non-motile, Gram-negative anaerobic bacterium that produces a range of virulence factors, particularly gingipains – arginine-C (RgpA and RgpB) or lysine-C (Kgp) specific cysteine proteinases [15, 16]. Gingipains are primarily located on the bacterial outer membrane but are also released into the extracellular environment in soluble form and via outer membrane vesicles (OMVs) [17]. As an asaccharolytic organism, *Pg* depends on gingipains for survival, growth and virulence as these proteases mediate host colonization, immune evasion, nutrient acquisition, and tissue degradation [15].

Given that gingipains have been shown to induce endothelial cell detachment and apoptosis, together with the cleavage of an adherence junction proteins e.g. VE-cadherin, it is possible that once *Pg* enters the bloodstream, it may compromise BBB integrity by targeting the cerebral endothelium [18], however, the mechanisms underlying *Pg* dissemination into the brain remain poorly understood.

Traditional *in vitro* models fail to fully mimic the structural and cellular complexity of the BBB and its dynamic interactions with microbial pathogens as they lack conditions such as flow and shear stress. In contrast, zebrafish (*Danio rerio*) have recently emerged as a valuable *in vivo* model for studying BBB physiology and pathology [19]. The zebrafish BBB shares strong structural and functional similarities with that of higher vertebrates, and transgenic zebrafish lines with fluorescently labeled vasculature allow real-time imaging of BBB function and disruption [19, 20].

Using a zebrafish larval model, we previously demonstrated that systemic *Pg* infection increases endothelial permeability and decreases cell surface abundance of endothelial adhesion molecules PECAM-1 and VE-cadherin in the intersegmental vessels and the caudal vein adjacent to the yolk sac [21]. In the present study, we extend these findings and focus our analysis on the effects of systemic *Pg* infection on BBB permeability and TJs expression on the cerebral vessels. We further examined the individual contribution of RgpA, RgpB, and Kgp gingipains to BBB disruption. Our data recognize gingipains as important mediators of the degradation of TJ proteins on the cerebral vessels, leading to pronounced BBB destruction, a phenomenon that may contribute to *Pg*-associated neurodegeneration. Therefore, these findings offer mechanistic insight into the neuroinflammatory potential of oral pathogens and may instruct development of future therapeutic strategies targeting BBB dysfunction in neurodegenerative diseases such as AD.

## Materials and methods

### Zebrafish husbandry, lines, and experimental conditions

Adult zebrafish were housed in aquaria equipped with a ZebTEC Stand Alone system (Tecniplast, Italy) under standard laboratory conditions (28 °C; 14-h light/10-h dark cycle) at the Zebrafish Core Facility of the Institute of Zoology and Biomedical Research, Jagiellonian University. The facility is licensed by the District Veterinary Inspectorate in Kraków (Ministry of Science and Higher Education, registration numbers 022 and 0057). Embryos were obtained from wild-type ABTL strain and from transgenic lines: *Tg(kdrl:mCherry-CAAX)*^*Y173*^ [22] and *Tg(fli1:EGFP)*^*y1*^ [23], both expressing fluorescent markers in endothelial cells.

Zebrafish larvae were maintained in E3 medium composed of 17.2 g/L NaCl (Chempur, Poland), 0.76 g/L KCl (POCH, Poland), 2.18 g/L CaCl₂ (EuroChem BGD, Poland), 2.4 g/L MgSO₄ (POCH, Poland), and supplemented with 0.00005% (w/v) methylene blue (Sigma-Aldrich, USA). Larvae were kept at 31 °C until 4 days post-fertilization (dpf). All experiments were performed in accordance with the European Community Council Directive 2010/63/EU. All procedures involving zebrafish larvae followed the ARRIVE guidelines.

### *Pg* strains and culture conditions

The wild-type *Pg* W83 and its isogenic gingipain-deficient mutants; gingipains-null (*ΔK/R-ab*), Kgp-null (*kgp*^Δ598−1732^::Tc^r^) and RgpA/RgpB-deficient (*rgpA*-::Cm^r^ *rgpB*^Δ410−507^::Em^r^) isogenic mutant strains [24] were maintained on tryptic soy agar (TSB; Sigma-Aldrich, USA) supplemented with 5 g/L yeast extract (BioShop, Canada), 5% (v/v) defibrinated sheep blood (Oxoid, UK), 0.25 g/L L-cysteine-HCl (BioShop, Canada), 5 mg/L hemin (Sigma-Aldrich, USA), and 0.5 mg/L menadione sodium bisulfite (Sigma-Aldrich, USA). The mutant strains were additionally cultured with 1 μg/mL tetracycline and/or 5 μg/mL erythromycin depending on the antibiotic cassette used to make the mutant. All bacteria were cultured anaerobically (80% N₂, 10% CO₂, 10% H₂) at 37 °C and prior to systemic microinjection into larvae, bacteria were transferred to liquid TSB supplemented with L-cysteine, hemin, and menadione and incubated overnight under anaerobic conditions. Cultures were then harvested by centrifugation (6,000 × g, 10 min), washed twice and resuspended in PBS (pH 7.4). Bacterial concentration was determined by measuring optical density at 600 nm (OD₆₀₀), with OD₆₀₀ = 7 corresponding to approximately 7 × 10⁹ colony-forming units (CFU)/mL, used for microinjection.

### Gingipain preparation and activation

Gingipains (RgpA, RgpB, and Kgp) were isolated and purified from *Pg* W83 culture supernatants, and their active concentrations were determined by active-site titration, as previously described [25]. Prior to systemic microinjection into larvae, each gingipain was activated by a twofold dilution in activation buffer (A-buffer) containing 0.2 M HEPES, pH 8.0 (Sigma-Aldrich, USA), 5 mM CaCl₂ (BioShop, Canada), and 20 mM L-cysteine (BioShop, Canada) freshly neutralized with 8 M NaOH (POCH, Poland), followed by incubation at 37 °C for 10 min [25].

### Systemic microinjection of *Pg* and gingipains into zebrafish larvae

Prior to microinjection, non-filament borosilicate glass capillaries were pulled to produce fine-tipped needles suitable for precise microinjections. Zebrafish larvae at 2 days post-fertilization (dpf) were anesthetized in E3 medium containing 160 mg/L tricaine (Sigma-Aldrich, USA) and systemically injected with 3 nL of either wild-type *Pg* W83 or *ΔK/R-ab* mutant suspensions at known concentrations into the common cardinal vein (duct of Cuvier) using a pneumatic PicoPump microinjector (World Precision Instruments, USA). An equal volume (3 nL) of sterile PBS was injected into control larvae. The concentration of injected bacteria was confirmed by plating serial dilutions of the bacterial suspension onto TSB anaerobic blood agar and enumerating CFUs after 5 days of incubation at 37 °C.

In a separate set of experiments, 3 nL of activated gingipains RgpA, RgpB or Kgp were injected into the duct of Cuvier of anesthetized 2 dpf zebrafish larvae. A corresponding volume of A-buffer was injected as a control. Gingipain concentrations were selected based on dose–response analyses performed in three independent experiments, involving 55–60 larvae per treatment group. For Kgp 0.4 μM, 0.5 μM and 0.6 μM, while for RgpA and RgpB, 0.6 μM, 1.2 μM and 1.6 μM concentrations were tested. Survival was assessed at selected time points by monitoring heartbeat activity. Morbidity was defined as the presence of pericardial edema. Active gingipain concentrations (0.6 μM for Kgp and 1.6 μM for both RgpA and RgpB), that induced morbidity without causing high mortality were selected for further experiments.

### Systemic microinjection of fluorescent tracers into zebrafish larvae

To assess BBB permeability, *Tg(kdrl:mCherry-CAAX) *^*Y173*^ zebrafish larvae were systemically microinjected with *Pg* W83 (wild-type or *ΔK/R-ab* mutant), gingipains (RgpA, RgpB, or Kgp), or appropriate control solutions (as described in "Systemic microinjection of Pg and gingipains into zebrafish larvae" section). Subsequently, fluorescent tracers were microinjected into the duct of Cuvier at defined time points to evaluate tracer leakage into the brain parenchyma. Two tracers with different molecular weights were used: 4’,6-diamidino-2-phenylindole, dihydrochloride (DAPI, 350 Da, Sigma–Aldrich, USA) and 70 kDa fluorescein isothiocyanate (FITC)-conjugated dextran (Sigma-Aldrich, USA). Both tracers were administered in 2 nL volume using a pneumatic PicoPump, from working solutions at concentrations of 2 mg/mL for DAPI and 25 mg/mL for FITC-dextran.

In larvae injected with gingipains, either DAPI or FITC-dextran was systemically administered 5 h after injection (hpi) of gingipains. In contrast, for larvae infected with bacteria, DAPI was systemically injected at 5 hpi, while FITC-dextran was systemically administered at 24 hpi. At the indicated time points, larvae were anesthetized and mounted laterally in 1% low-melting-point agarose in E3 medium on glass-bottomed imaging dishes. Confocal imaging was performed using a Zeiss LSM 900 microscope equipped with an Airyscan 2 detector and 20 × NA 0.80 objective. A total of 10–15 larvae per group were analyzed, including those injected with wild-type *Pg* W83, the *ΔK/R-ab* mutant, PBS, RgpA, RgpB, Kgp, or A-buffer. Maximum intensity Z-stack projections were generated for each larva. DAPI leakage was quantified by measuring both the total area and mean fluorescence intensity of the tracer signal within the brain parenchyma. For FITC-dextran, mean fluorescence intensity was measured both inside and outside cerebral blood vessels, and a fluorescence intensity ratio was calculated to assess tracer leakage. The number of visible cerebral vessels was enumerated, and the mean vessel diameter was determined based on the mCherry fluorescence signal. Vessel patency was assessed by calculating the percentage of vessels showing green fluorescence localized within the vascular lumen, corresponding to FITC-conjugated dextran inside mCherry-positive cerebral vasculature.

### Whole-mount immunohistochemical staining of zebrafish larvae

To visualize the localization of Claudin-5 and Zo-1 within the BBB of *Tg(fli1:EGFP)*^*y1*^ zebrafish larvae, whole-mount immunohistochemical (IHC) staining was performed on fixed larval heads. At the indicated experimental time points, larvae were euthanized in E3 medium supplemented with tricaine (160 mg/L; Sigma-Aldrich, USA) and fixed overnight at 4 °C in 4% (w/v) paraformaldehyde (PFA; Sigma-Aldrich, USA) prepared in PBS (pH 7.4).

Following fixation, larval heads were dissected under a stereomicroscope. Twelve heads per experimental group were pooled into a single microcentrifuge tube and washed three times for 20 min in PBS (pH 7.4) on a rotator shaker at room temperature. Samples were then washed three times for 10 min each in PBST (PBS containing 0.1% Tween-20; Sigma-Aldrich, USA), dehydrated through a graded methanol series (25%, 50%, 75%, and 100%; Sigma-Aldrich, USA), and stored in 100% methanol at −20 °C overnight.

For immunostaining, samples were rehydrated through decreasing concentrations of methanol back to PBST and washed for 10 min on a rotator shaker. Permeabilization was performed using proteinase K (10 µg/mL; Invitrogen, USA) for 15 min at room temperature, followed by three 5-min washes in PBST. Samples were then incubated in ice-cold acetone (POCH, Poland) at −20 °C for 6 min, washed again in PBST (3 × 5 min), and blocked in 10% bovine serum albumin (BSA; Sigma-Aldrich, USA) in PBST for 3 h at room temperature on a rotator shaker. Primary antibody incubation was performed overnight at room temperature with either mouse anti-Claudin-5 (4C3C2; Invitrogen, USA; 1:250) or mouse anti-Zo-1 (1A12; Invitrogen, USA; 1:250), both diluted in blocking buffer. After incubation, samples were washed three times for 30 min each in PBST. Secondary antibody incubation was conducted overnight at room temperature using Alexa Fluor 647-conjugated goat anti-mouse IgG (A21235; Invitrogen, USA, 1:500 in blocking buffer), followed by three 30-min washes in PBST and a final rinse in PBS (pH 7.4) to remove residual detergent.

Confocal imaging was carried out using a Zeiss LSM 900 microscope equipped with an Airyscan 2 detector and both 20 × NA 0.80 and 40 × NA 1.2 water-immersion objectives. For each condition, 9–12 larval heads were imaged. Z-stack images were acquired, and maximum intensity projections were generated using ZEN software (Carl Zeiss Microscopy GmbH, Germany). Brain vasculature was manually delineated based on EGFP fluorescence, and the mean fluorescence intensities of Claudin-5 and Zo-1 immunoexpression within the outlined cerebral vessel areas were measured.

### Western blot analysis of TJ protein level

At 24 h post administration of gingipains (RgpA, RgpB, Kgp) or *Pg* (ΔK/R-ab, W83), the heads of zebrafish larvae were separated and homogenized in RIPA lysis buffer (20 heads per sample). Total proteins were isolated, and their concentrations were quantified using the bicinchoninic acid (BCA) assay. Equal amounts of protein (20 µg per lane) were resolved by SDS-PAGE and subsequently transferred onto PVDF membranes. Following transfer, membranes were washed and blocked for 1 h in 5% non-fat milk. They were then incubated overnight at 4 °C with primary antibodies against Zo-1 (Invitrogen, USA; ZO1-1A12; 1:1000) or claudin-5 (Invitrogen, USA; 4C3C2; 1:1000). After extensive washing, membranes were exposed to horseradish peroxidase (HRP)-conjugated secondary antibodies (Cell Signaling Technology, USA; 7076S; 1:10,000) for 1 h under gentle agitation. Protein detection was performed using the SuperSignal™ West Atto Ultimate Sensitivity Substrate (Thermo Fisher Scientific, USA) and ChemiDoc XRS + System (BioRad Laboratories, CA, USA). To verify equal protein loading, membranes were stripped using a low-pH buffer for 30 min and probed again with an anti-β-actin antibody (Sigma-Aldrich, USA; A5316, 1:1000).

### RNA isolation and cDNA synthesis

Wild-type ABTL zebrafish larvae systemically microinjected with wild-type *Pg* W83, the *ΔK/R-ab* mutant, or PBS, as well as with gingipains (RgpA, RgpB, Kgp) or A-buffer, were anesthetized with tricaine at the indicated time points. Larval heads were separated from the trunks using a sterile scalpel, and brains were carefully isolated under a stereomicroscope. Thirteen larval brains were pooled per sample. Total RNA was extracted from zebrafish larval brains using TRIzol™ Reagent (Invitrogen, USA) according to the manufacturer’s instructions. RNA concentration and purity were assessed spectrophotometrically using a microplate reader (Tecan Group Ltd., Switzerland). To remove potential genomic DNA contamination, 1 U of RNase-free DNase (Promega, USA) was applied following the manufacturer's protocol. The synthesis of cDNA was performed using 100 ng of total RNA and the High-Capacity cDNA Reverse Transcription Kit (Applied Biosystems, USA), according to the manufacturer’s instructions. No-reverse transcriptase (no-RT) controls were included. Synthesized cDNA was diluted 20-fold in nuclease-free water (EURx, Poland) and stored at − 20 °C until use in real-time quantitative PCR (RT-qPCR). Two independent experiments were conducted, each including 2–3 biological replicates.

### Real-Time qPCR

Quantitative gene expression analysis was performed on cDNA templates using Rotor-Gene Q (Qiagen, Hilden, Germany), following the procedure described by Rakus et al. [26]. Each qPCR reaction was carried out in duplicate, with no-reverse transcriptase (no-RT) and non-template controls (NTC) included. Target gene expression was normalized to the reference gene *rps11* (ribosomal protein s11) and quantified using the Pfaffl method [27], according to the following equation:

$$Ratio=\frac{{({E}_{target})}^{\Delta Ct}{Target}^{(control-sample)}}{{({E}_{reference})}^{\Delta Ct}{Reference}^{(control-sample)}}$$

where E is the amplification efficiency, and Ct is the threshold cycle. The primer sequences for the assessed genes *cld5a*, *cld5b*, *tjp1a*, and *tjp1b* were previously published by Pellegrini et al. [20], and the primer sequences for the reference gene *rps11* were described by Rakus et. al. [26].

### Statistical analysis

Statistical significance of differences in Claudin-5 and Zo-1 immunoexpression was assessed using one-way ANOVA followed by Tukey’s post hoc test. Changes in gene expression, fluorescent tracer leakage, and the number and morphology of cerebral vessels were analyzed using two-way ANOVA with Sidak’s multiple comparisons test. All statistical analyses were performed in GraphPad Prism version 9.5.1 (GraphPad Software Inc., USA). Data are presented as mean ± standard deviation (SD). Differences were considered statistically significant at *p* ≤ 0.05.

## Results

### BBB impairment induced by systemic *Pg* infection

#### Increased BBB permeability and cerebrovascular alterations upon systemic *Pg* infection

BBB permeability was assessed in transgenic zebrafish larvae *Tg(kdrl:mCherry-CAAX) *^*Y173*^ systemically infected with either the wild-type *Pg* W83 strain or the gingipain-deficient mutant *ΔK/R-ab*, along with PBS-injected controls. Larvae were microinjected with fluorescent tracers of different molecular sizes: DAPI (350 Da) and FITC-conjugated dextran (70 kDa).

**Fig. 1:**
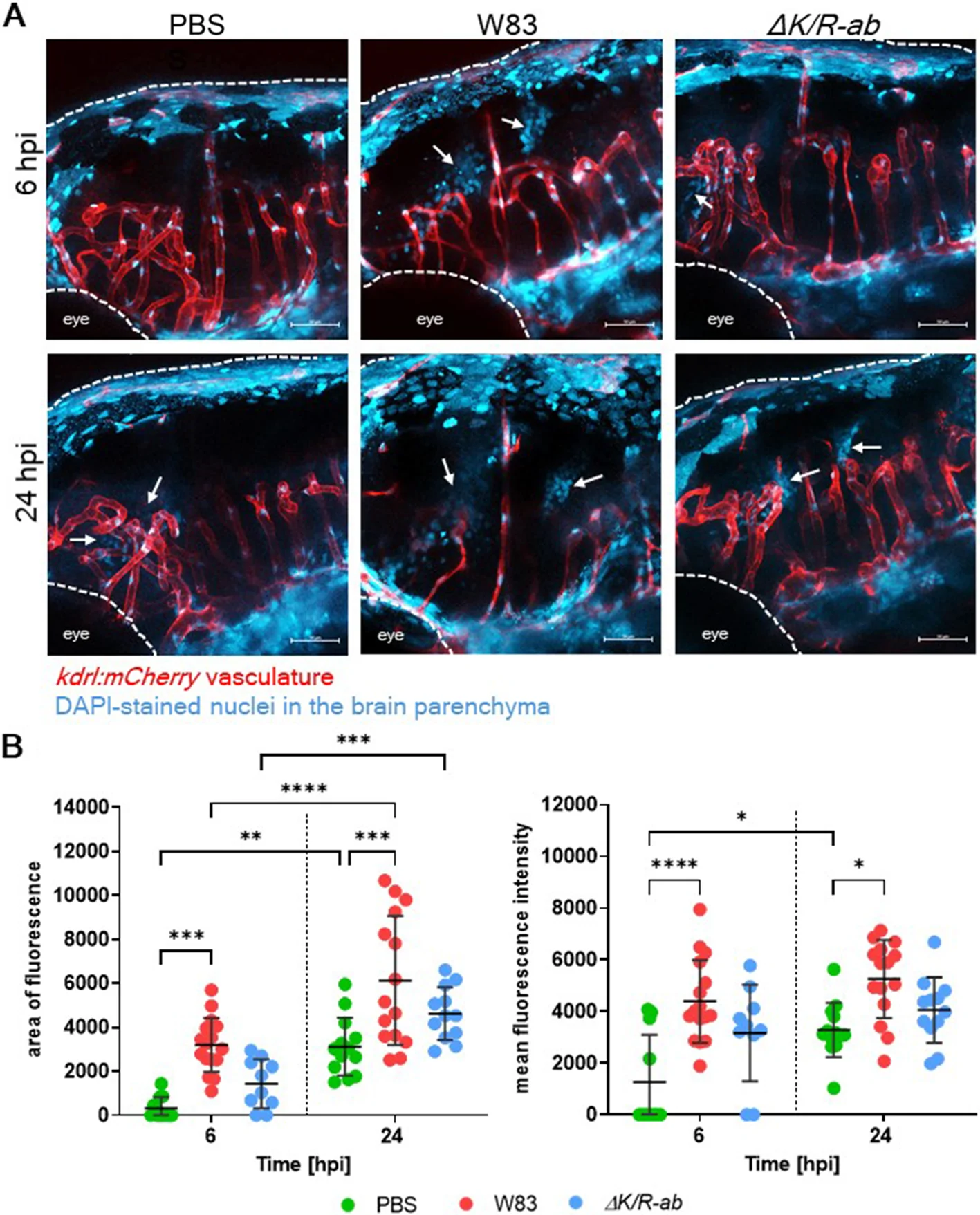
Systemic infection with wild-type *P. gingivalis*, but not the gingipain-deficient mutant *ΔK/R-ab*, increases BBB permeability at early infection stages. Transgenic zebrafish larvae *Tg(kdrl:mCherry-CAAX)*.^*Y173*^, with fluorescently labeled vasculature, were systemically injected with *P. gingivalis* W83, the *ΔK/R-ab* mutant, or PBS at 3 days post fertilization (dpf). To assess early BBB leakage, 5 h post bacterial infection larvae were injected with 350 Da DAPI. Real-time *in vivo* confocal imaging was performed at 6 and 24 h post injection (hpi). **A** Representative confocal images of the larval heads show endothelial cell nuclei stained with DAPI in PBS-, W83-, and *ΔK/R-ab*-injected larvae. DAPI leakage from brain vessels into the surrounding parenchyma is indicated with white arrows. Vasculature is labeled in red (*kdrl:mCherry*). **B** Quantification of BBB leakage based on DAPI-stained fluorescent area and DAPI mean fluorescence intensity in the brain parenchyma. Data are presented as mean ± SD (*n* = 10–15 larvae per group). Each dot represents one larva. Data were obtained from at least three independent experiments. Statistical analysis was performed using two-way ANOVA with Sidak’s multiple comparisons test. *p ≤ 0.05, **p ≤ 0.01, ***p ≤ 0.001, ****p ≤ 0.0001

To examine early and late BBB leakage, DAPI extravasation was analyzed at 6 and 24 hpi, respectively. Already at 6 hpi, DAPI leakage into the surrounding parenchyma was clearly visible in larvae infected with *Pg* W83, with this tracer accumulation mainly near the primary vertical brain vessels. In contrast, *ΔK/R-ab*-infected and control larvae showed DAPI signal only in the nuclei within the healthy endothelial cells, with physiologically minimal or no leakage into the brain tissue (Fig. 1A). At 24 hpi, DAPI extravasation became detectable in the brain parenchyma of both *ΔK/R-ab*-infected and control larvae due to its low molecular weight, but still tracer diffusion remained more extensive upon *Pg* W83 infection. Quantitative analysis confirmed that both the leakage area and mean fluorescence intensity were significantly elevated in *Pg* W83-infected larvae compared to control ones at 6 and 24 hpi, whereas in *ΔK/R-ab*-infected larvae did not differ significantly from control ones (Fig. 1B).

**Fig. 2:**
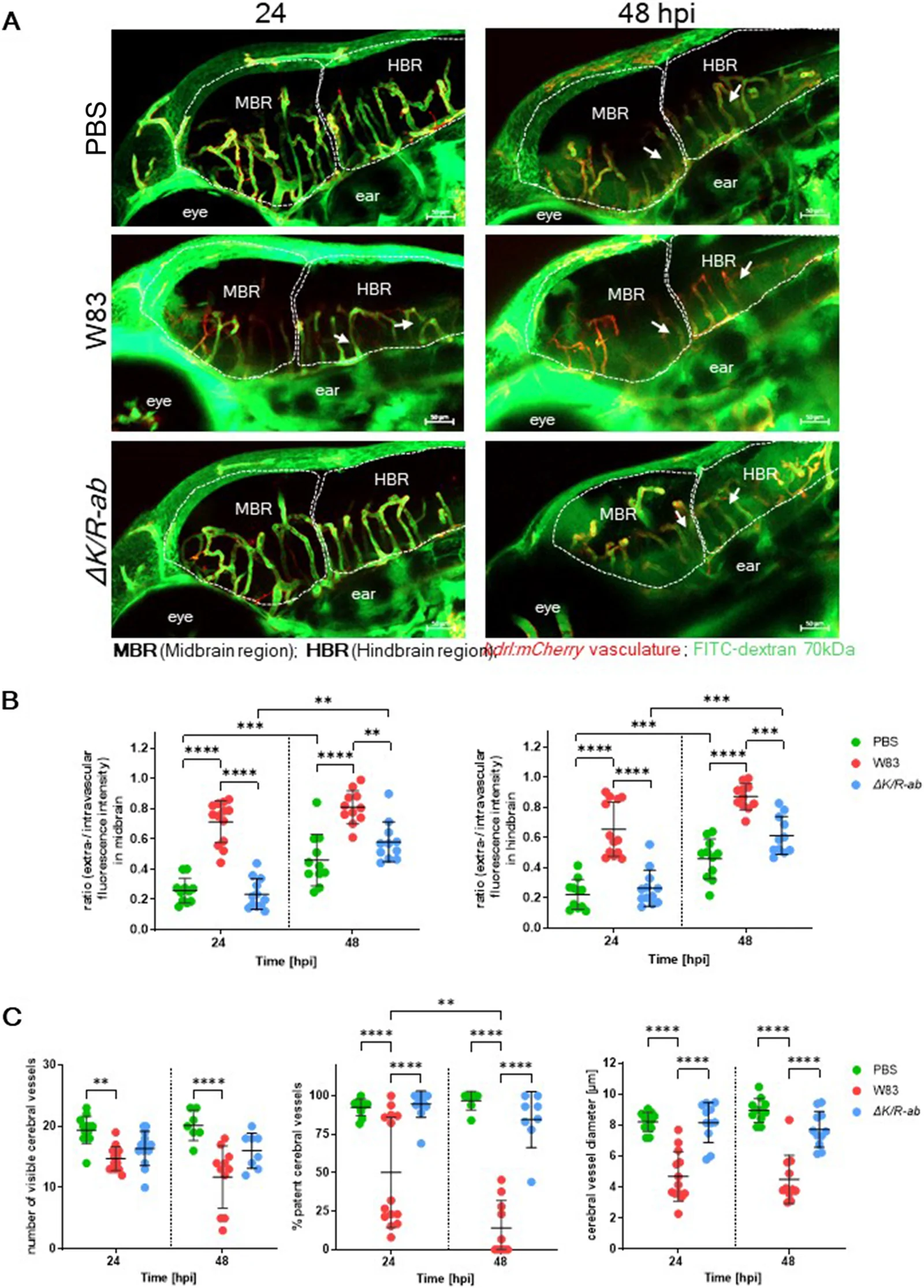
Systemic *P. gingivalis* infection leads to further BBB impairment at late infection stages. Transgenic zebrafish larvae *Tg(kdrl:mCherry-CAAX)*.^*Y173*^, with fluorescently labeled vasculature, were systemically injected with *P. gingivalis* W83, the *ΔK/R-ab* mutant, or PBS at 3 days post fertilization (dpf). To assess BBB leakage, 24 h post bacterial infection larvae were injected with 70 kDa FITC-dextran. Real-time *in vivo* confocal imaging was performed at 24 and 48 h post injection (hpi). **A** Representative confocal images of the larval heads show FITC-dextran leakage (green), indicated by white arrows, from brain vessels into the surrounding parenchyma of the midbrain (MBR) and hindbrain (HBR) regions, outlined with dashed lines, in PBS-, W83-, and *ΔK/R-ab*-injected larvae. Vasculature is labeled in red (*kdrl:mCherry*). Morphological changes in cerebral vessels, including reduced vessel number, vessel narrowing and loss of patency were observed in response to systemic infection with *P. gingivalis* W83, but not upon infection with *ΔK/R-ab*. **B** Quantification of FITC-dextran leakage into the brain parenchyma, expressed as the ratio of fluorescence intensity outside versus inside the vessels in the MBR and HBR regions. **C** Quantification of cerebral vessel changes, including the number of visible vessels, percentage of patency, and mean cerebral vessel diameter. Data are presented as mean ± SD (*n* = 10–15 larvae per group). Each dot represents one larva. Data were obtained from at least three independent experiments Statistical analysis was performed using two-way ANOVA with Sidak’s multiple comparisons test. **p ≤ 0.01, ***p ≤ 0.001, ****p ≤ 0.0001

To assess BBB permeability at later infection stages, we analyzed 70 kDa FITC-dextran leakage. At 24 hpi, slight dextran diffusion was observed in the midbrain and hindbrain of larvae infected with the *Pg* W83, while dextran remained retained within cerebral vessels in control and *ΔK/R-ab*-infected larvae. By 48 hpi, dextran extravasation appeared in control and *ΔK/R-ab*-infected larvae, but was markedly less extensive than upon *Pg* W83 infection (Fig. 2A). Quantitative measurement of dextran leakage, expressed as the ratio of fluorescence intensity outside versus inside the vessel, confirmed significantly higher tracer diffusion in *Pg* W83-infected larvae in midbrain and hindbrain regions at both time points compared to control and *ΔK/R-ab*-infected larvae (Fig. 2B). The forebrain region was excluded from analysis due to high background signal from skin vessels, which are naturally more permeable.

In addition to tracer leakage, we assessed detailed changes in the brain vasculature. Firstly, the total number of visible cerebral vessels was quantified. Larvae infected with the *Pg* W83 strain exhibited a significant reduction in the number of visible vessels compared to control and *ΔK/R-ab*-infected larvae at both examined time points (Fig. 2C, left panel). Secondly, we analyzed the percentage of vasoconstricted vessels and observed that systemic infection with the *Pg* W83 strain led to a marked decrease in vessel patency, dropping by about 50% at 24 hpi and further declining to approximately 15% at 48 hpi. In contrast, the patency of cerebral vessels remained unchanged in *ΔK/R-ab*-infected larvae compared to control (Fig. 2C, middle panel). Finally, the mean diameter of the remaining patent vessels was measured. *Pg* W83 infection resulted in a significant narrowing of cerebral vessels compared to controls, whereas infection with the *ΔK/R-ab* mutant had no significant effect on vessel lumen (Fig. 2C, right panel).

Taken together, these results demonstrate that systemic *Pg* infection, in a gingipain-dependent manner, disrupts BBB integrity, promotes leakage of both small (350 Da DAPI) and large (70 kDa FITC-conjugated dextran) tracers into the brain parenchyma, and induces marked changes in cerebral vessel number, patency and morphology.

#### Reduced TJ protein expression in cerebral vessels following *Pg* infection

**Fig. 3:**
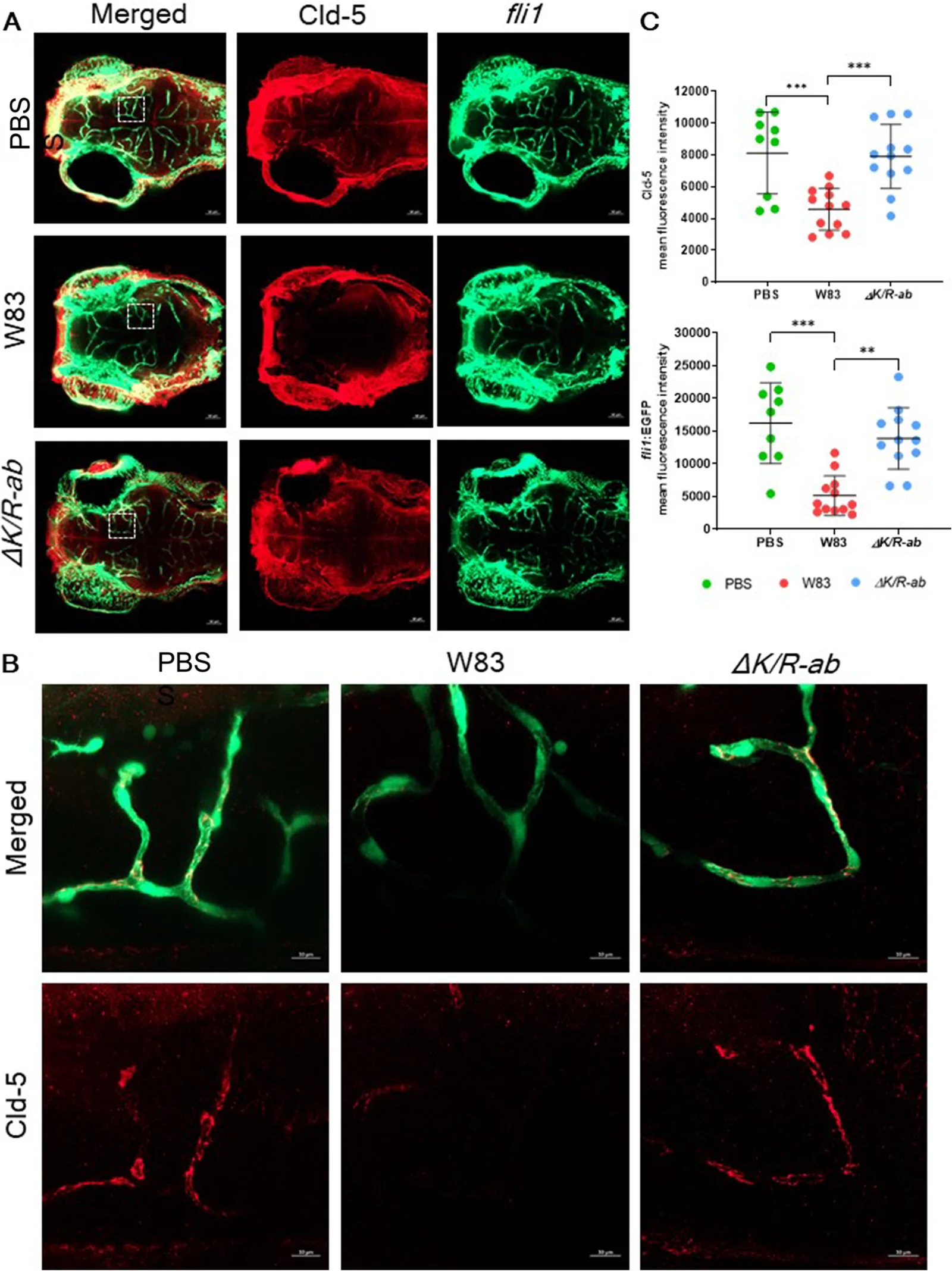
Systemic infection with *P. gingivalis*, but not the gingipain-deficient mutant *ΔK/R-ab*, reduces Claudin-5 expression within the cerebral vessels. Transgenic zebrafish larvae *Tg(fli1:EGFP)*.^*y1*^, with fluorescently labeled vasculature, were systemically injected with *P. gingivalis* W83, the *ΔK/R-ab* mutant, or PBS at 3 days post fertilization (dpf). Changes in Claudin-5 expression were assessed by IHC staining performed on fixed larval heads at 24 h post injection (hpi). **A** Representative confocal images show Claudin-5 immunoexpression (red) within cerebral vasculature (green) in PBS-, W83- and *ΔK/R-ab*-injected larvae. **B** Enlarged regions correspond to white dashed boxes shown in (A). **C** Quantification of mean fluorescence intensity corresponding to Claudind-5 labelling and EGFP signal within cerebral vessels. Data are presented as mean ± SD (*n* = 9–12 larvae per group). Each dot represents one larva. Data were obtained from at least two independent experiments. Statistical analysis was performed using one-way ANOVA followed by Tukey’s post hoc test, **p ≤ 0.01, ***p ≤ 0.001

To assess whether the increased leakage of fluorescent tracers into the brain parenchyma upon systemic *Pg* W83 infection results from tight junction (TJ) protein degradation, we performed immunolabeling of Claudin-5 and Zo-1 in the heads of *Tg(fli1:EGFP)*^*y1*^ zebrafish larvae, which express EGFP specifically in endothelial cells. At 24 hpi, in control larvae and those infected with the *ΔK/R-ab* mutant, Claudin-5 immunoreactivity was strongly colocalized with EGFP-positive cerebral vessels (Fig. 3A). In contrast, in the brain of the larvae infected with the *Pg* W83 strain, Claudin-5 labelling was detected only in single vessel segments. *In vivo* imaging at higher-magnification further confirmed strong Claudin-5 labelling overlapping with EGFP signal in control and *ΔK/R-ab*-infected larvae, whereas in *Pg* W83-infected larvae, Claudin-5 was weakly detected and appeared only along short vessel sections (Fig. 3B).

Quantitative analysis of mean fluorescence intensity of Claudin-5 immunoexpression within the brain vasculature showed a significant reduction in *Pg* W83-infected larvae compared to both control and *ΔK/R-ab*-infected ones (Fig. 3C). In contrast, infection with the *ΔK/R-ab* mutant did not affect Claudin-5 expression compared to control larvae. Additionally, systemic infection with *Pg* W83, but not the *ΔK/R-ab* mutant, significantly decreased mean EGFP fluorescence intensity in cerebral vessels, supporting our observations from *Tg(kdrl:mCherry-CAAX) *^*Y173*^ larvae, where vessel degradation in response to *Pg* W83 infection was evident.

**Fig. 4:**
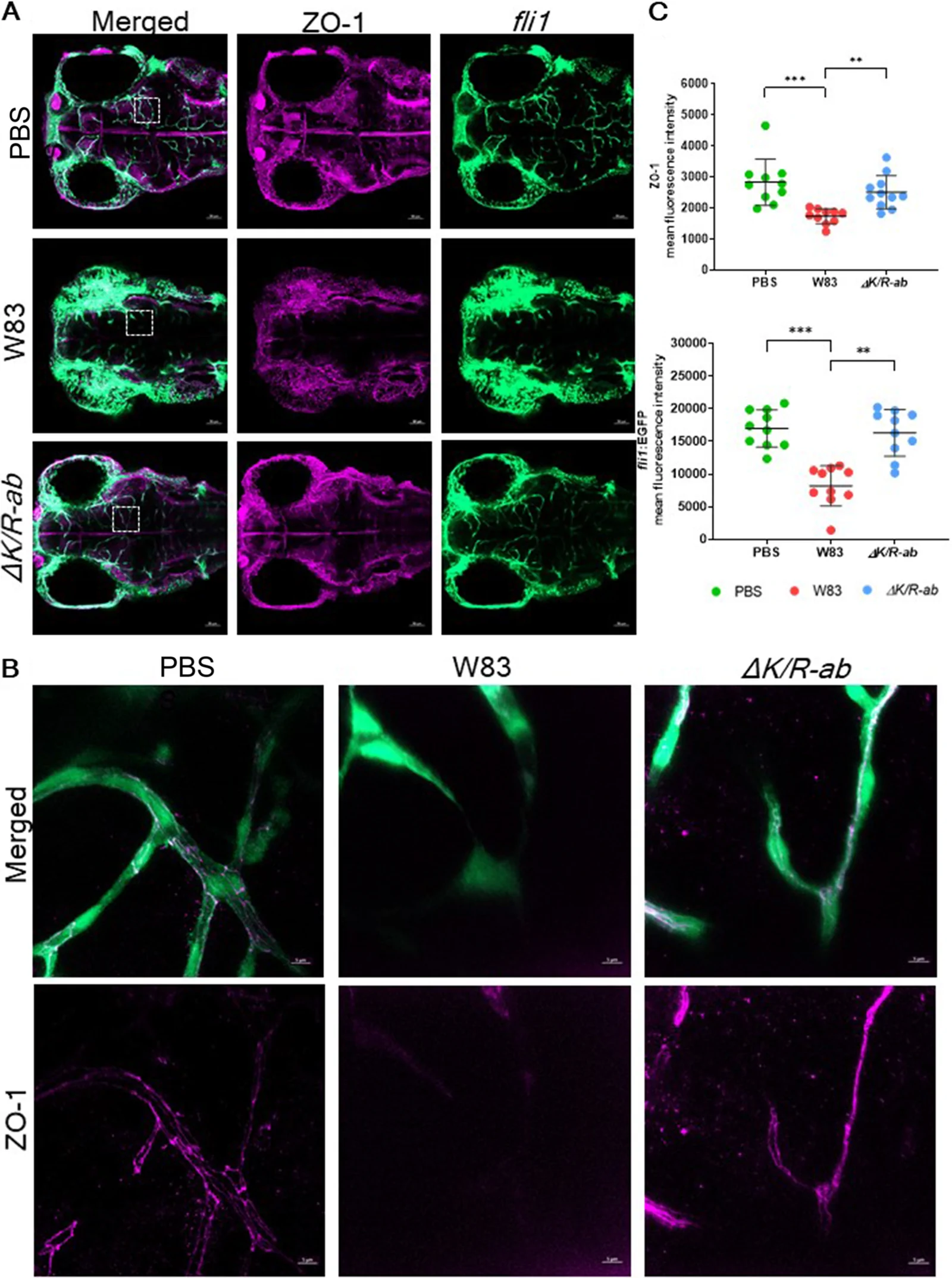
*P. gingivalis* decreases Zo-1 expression within the cerebral vessels. Transgenic zebrafish larvae *Tg(fli1:EGFP)*.^*y1*^, with fluorescently labeled vasculature, were systemically injected with *P. gingivalis* W83, *the ΔK/R-ab* mutant, or PBS at 3 days post fertilization (dpf). Zebrafish larvae were than fixed at 24 h post injection (hpi) and IHC labelling was performed to visualize Zo-1 immunoexpression. **A** Representative confocal images show Zo-1 labelling (magenta) within cerebral vasculature (green) in PBS-, W83- and *ΔK/R-ab*-injected larvae. **B** Enlarged regions correspond to white dashed boxes shown in (A). **C** Quantification of mean fluorescence intensity corresponding to Zo-1 labelling and EGFP signal within brain vasculature. Data are presented as mean ± SD (*n* = 9–12 larvae per group). Each dot represents one larva. Data were obtained from at least two independent experiments. Statistical analysis was performed using one-way ANOVA followed by Tukey’s post hoc test, **p ≤ 0.01, ***p ≤ 0.001

To further characterize TJ changes, IHC staining for Zo-1 was performed. Similar to claudin-5, at 24 hpi Zo-1 immunoreactivity within cerebral vessels was markedly reduced in *Pg* W83-infected larvae compared to control and *ΔK/R-ab*-infected ones, while control and *ΔK/R-ab*-infected larvae showed comparable Zo-1 immunoexpression (Fig. 4A and B). Quantification of mean fluorescence intensity Zo-1 immunoexpression confirmed these findings (Fig. 4C). Notably, in zebrafish larvae immunolabeled for Zo-1, we again observed that *Pg* W83 infection led to a significant decrease in mean EGFP fluorescence intensity within cerebral vasculature compared to both control and *ΔK/R-ab*-infected larvae, similar to what was seen with claudin-5 staining.

We also performed Western blot analysis of Zo-1 and Claudin-5 levels in the larval heads and found that *Pg* W83, but not *Pg ΔK/R-ab*, reduced the level of Zo-1. Similar pattern was not observed for Claudin-5 which levels were comparable in the heads of PBS-, *Pg* W83- and *Pg ΔK/R-ab-i*njected larvae (Supplementary Fig. 1S).

Overall, these results strongly suggest a key role of gingipains in TJ protein degradation and BBB disruption.

#### TJ gene expression remains unchanged upon systemin *Pg* infection

To evaluate whether decreased level of TJ proteins in *Pg* W83-infected larvae could be also an effect of downregulated expression of genes encoding these proteins, we investigated whether systemic *Pg* infection affects TJ transcriptional levels, which could contribute to BBB dysfunction. In zebrafish, two paralogs of the claudin-5 gene (*cldn5a* and *cldn5b*) and two paralogs of the zonula occludens-1 gene (*tjp1a* and *tjp1b*) have been identified and were included in our analysis. Neither systemic infection with the *Pg* W83 strain nor with the *ΔK/R-ab* mutant significantly affected the expression of *cldn5a*, *cldn5b*, *tjp1a*, or *tjp1b* in larval brains compared to PBS-injected controls (Supplementary Fig. 2S). The expression levels of these genes remained unchanged relative to control at both time points examined (24 and 48 hpi), indicating that the impact of *Pg* on BBB integrity likely results from direct degradation of TJ proteins rather than their transcriptional downregulation.

### Gingipains are involved in BBB disruption

#### Gingipains affect BBB permeability and cerebrovascular changes

**Fig. 5:**
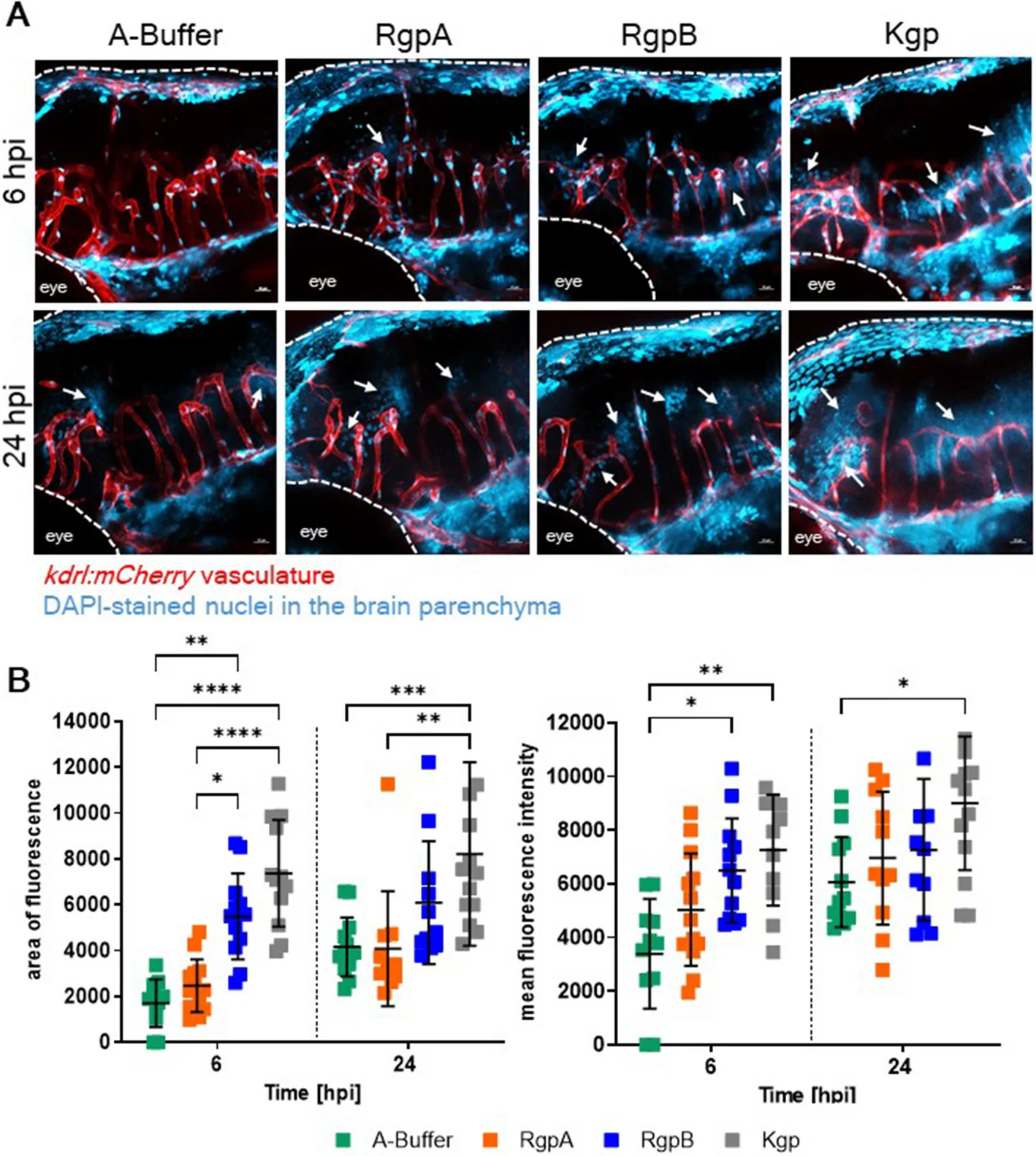
Systemically injected gingipains RgpB and Kgp, but not RgpA, selectively increase BBB permeability to the small-molecular-weight tracer DAPI (350 Da). Transgenic zebrafish larvae *Tg(kdrl:mCherry-CAAX)*.^*Y173*^, with fluorescently labeled vasculature (red), were systemically injected with RgpA, RgpB, Kgp, or A-buffer at 3 days post fertilization (dpf). To assess BBB leakage, larvae were injected with 350 Da DAPI 5 h after gingipain administration. Real-time *in vivo* confocal imaging was performed at 6 and 24 h post injection (hpi). **A** Representative confocal images of the larval heads show endothelial cell nuclei stained with DAPI in larvae injected with RgpA, RgpB, Kgp, or A-buffer. DAPI diffusion from brain vessels into the surrounding parenchyma is indicated by white arrows. **B** Quantification of BBB leakage based on the DAPI-stained fluorescent area and mean fluorescence intensity in the brain parenchyma. Data are presented as mean ± SD (*n* = 10–15 larvae per group). Each dot represents one larva. Data were obtained from at least three independent experiments. Statistical analysis was performed using two-way ANOVA with Sidak’s multiple comparisons test. *p ≤ 0.05, **p ≤ 0.01, ***p ≤ 0.001, ****p ≤ 0.0001

Since the wild-type *Pg* W83 strain caused clear BBB disruption, whereas the *ΔK/R-ab* mutant did not, we further investigated the impact of individual gingipains (RgpA, RgpB, and Kgp) on the BBB integrity and cerebral vessel morphology. *Tg(kdrl:mCherry-CAAX) *^*Y173*^ zebrafish larvae were systemically injected with each gingipain at doses determined by preliminary dose–response survival analyses, which showed clear concentration-dependent effects on larval survival (Supplementary Fig. 3S). The selected doses induced BBB alterations while minimizing larval mortality. DAPI (350 Da) and FITC-conjugated dextran (70 kDa) were used to assess BBB permeability at 6 and 24 hpi.In larvae treated with all three gingipains visible DAPI leakage into the brain parenchyma as early as 6 hpi was observed, with dye accumulation near major cerebral vessels. In contrast, A-buffer-injected control showed DAPI staining restricted to endothelial cell nuclei, with no signs of extravasation (Fig. 5A). By 24 hpi, tracer diffusion markedly increased in larvae treated with RgpB and Kgp, whereas the control and RgpA-injected larvae showed minimal leakage of cerebral vessels. Quantitative analysis revealed that at 6 hpi, the leakage area was significantly larger in RgpB- and Kgp-injected larvae compared to control and RgpA-treated ones (Fig. 5B). At the later time point, a significant increase in DAPI leakage was observed only in the Kgp-injected larvae relative to control and RgpA-treated ones. Similarly, at 6 hpi DAPI mean fluorescence intensity was significantly higher in the brain area of RgpB- and Kgp-treated larvae and remained elevated in the Kgp-injected larvae at 24 hpi.

**Fig. 6:**
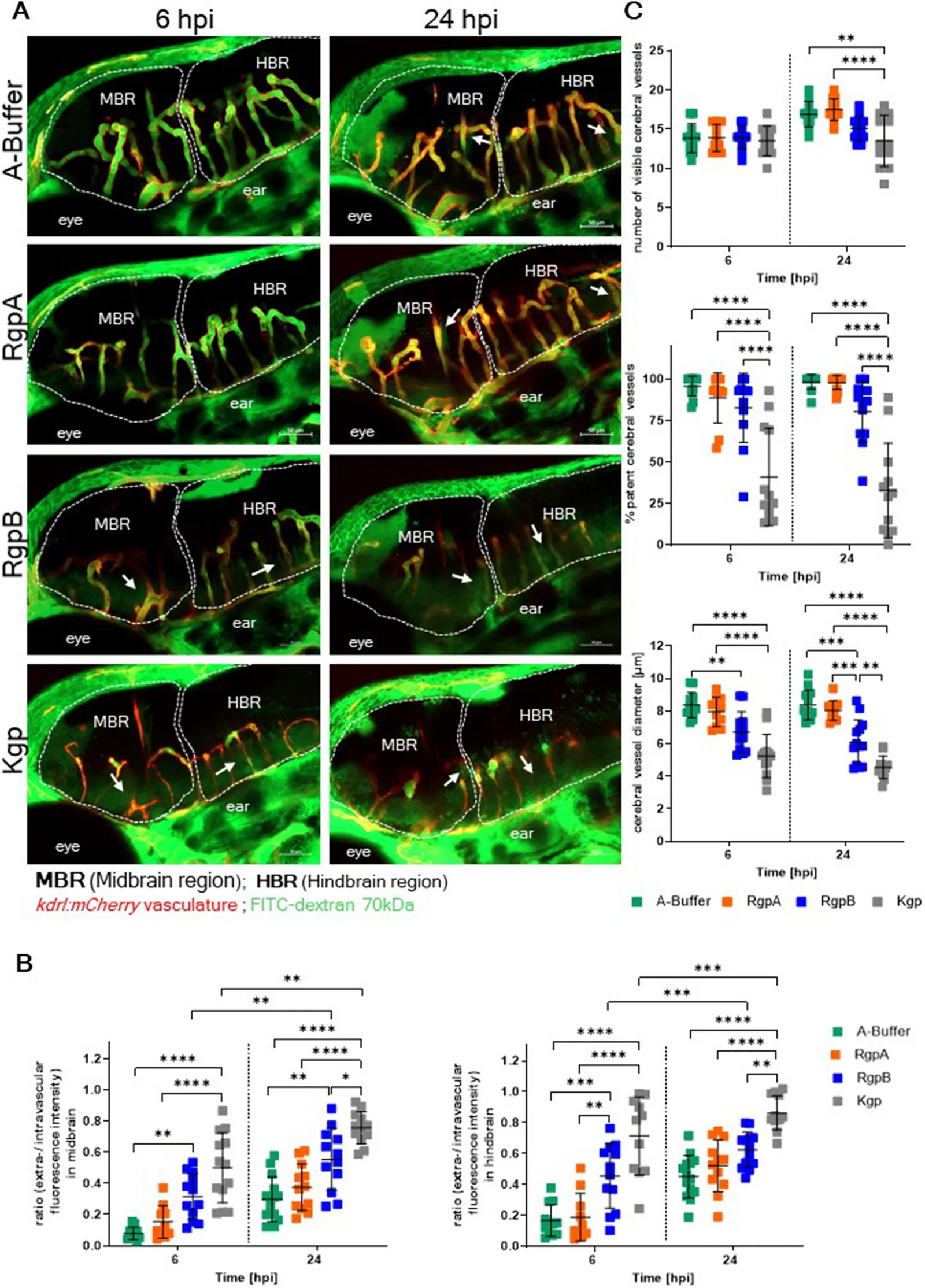
Systemically injected gingipains RgpB and Kgp, but not RgpA, also induce severe BBB disruption visualized by leakage of large-molecular-weight FITC-dextran (70 kDa). Transgenic zebrafish larvae *Tg(kdrl:mCherry-CAAX)*.^*Y173*^, with fluorescently labeled vasculature (red), were systemically injected with RgpA, RgpB, Kgp, or A-buffer at 3 days post fertilization (dpf). To examine BBB disruption, larvae were injected with 70 kDa FITC-dextran 5 h post gingipain administration. Real-time* in vivo* confocal imaging was performed at 6 and 24 h post injection (hpi). **A** Representative confocal images of the larval heads show FITC-dextran leakage (green), indicated by white arrows, from cerebral vessels into the surrounding parenchyma of the midbrain (MBR) and hindbrain (HBR) regions, outlined with dashed lines, in RgpA-, RgpB-, Kgp- and A-buffer-injected larvae. Morphological changes within cerebral vasculature including reduced vessel number, vessel narrowing and loss of patency are visible. **B** Quantification of FITC-dextran leakage into the brain parenchyma, expressed as the ratio of fluorescence intensity outside versus inside the vessels in the MBR and HBR regions. **C** Quantification of cerebral vessel changes, with the number of visible vessels, percentage of patency, and mean cerebral vessel diameter. Data are presented as mean ± SD. Each dot represents one larva. Data were obtained from at least three independent experiments (*n* = 10–15 larvae per group). Statistical analysis was performed using two-way ANOVA with Sidak’s multiple comparisons test. *p ≤ 0.05, **p ≤ 0.01, ***p ≤ 0.001, ****p ≤ 0.0001

Consistent with the DAPI results, FITC-dextran leakage confirmed that gingipain injection affected BBB permeability. At 6 hpi, slight dextran diffusion was detected in the midbrain and hindbrain of RgpB- and Kgp-treated larvae, whereas in control and RgpA-injected larvae the tracer was retained within cerebral vessels (Fig. 6A). By 24 hpi, dextran extravasation markedly increased in RgpB- and Kgp-injected larvae, while remaining low in control and RgpA-injected ones. Quantification of the fluorescence intensity ratio (outside vs. inside vessels) confirmed significantly higher values for RgpB- and Kgp-treated larvae at both studied time points and in both brain regions (Fig. 6B). Notably, leakage after Kgp injection was also significantly higher than that observed in RgpA-treated larvae among the whole larval brain at all examined time points.

In addition to increased BBB leakage, gingipain injection altered cerebral vessel morphology. At 24 hpi, the total number of visible vessels was significantly reduced in Kgp-treated larvae compared to A-buffer-injected and RgpA-treated ones (Fig. 6C, upper panel). Moreover, at 6 hpi we observed decreased vessel patency by about 60% in the larvae from the Kgp group and it declined further by 24 hpi, whereas it remained unchanged in control larvae and those injected with RgpA and RgpB (Fig. 6C, middle panel). Finally, at both time points the mean diameter of remaining patent vessels was significantly narrower in Kgp- and RgpB-injected larvae compared to control and RgpA ones (Fig. 6C, lower panel). Among the tested proteases, Kgp consistently induced the most pronounced effects on both BBB permeability and cerebral vessel morphology.

Together, these findings suggest that systemic administration of gingipains, in particular Kgp, either directly or indirectly by activating of yet unknown host pathways, enhances parenchymal leakage of both small and large fluorescent tracers, and induces significant changes in cerebral vessel number, patency, and morphology, underscoring their key role in *Pg*-mediated BBB damage.

#### Gingipains contribute to TJ protein loss in cerebral vessels

**Fig. 7:**
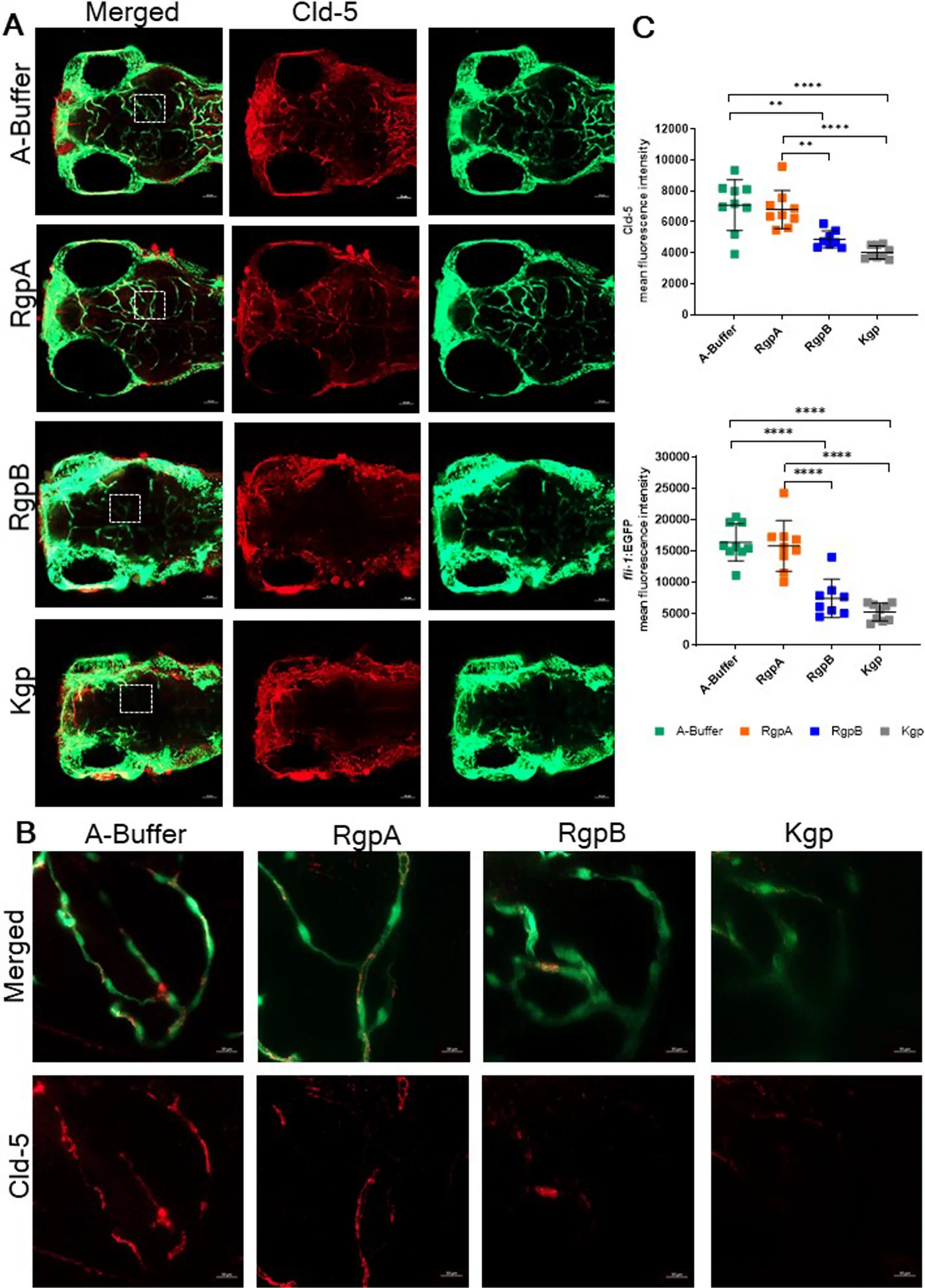
Claudin-5 protein expression within BBB is reduced in response to systemic injection of RgpB and Kgp, but not RgpA. Transgenic *Tg(fli1:EGFP)*.^*y1*^ zebrafish larvae with fluorescently labeled vasculature (green) were systemically injected with RgpA, RgpB, Kgp, or A-buffer at 3 days post fertilization (dpf). Claudin-5 protein expression was assessed by IHC staining of fixed larval heads collected at 24 h podt injection (hpi). **A** Representative confocal images show Claudin-5 immunoexpression (red) within cerebral vasculature in RgpA- RgpB-, Kgp-, or A-buffer-injected larvae. **B** Magnified representative regions indicated by white dashed boxes in (**A**). **C** Quantification of Claudin-5 and EGFP mean fluorescence intensity within cerebral vessels. Each dot represents one larva. Data are presented as mean ± SD (*n* = 9–12 larvae per group). Data were obtained from at least two independent experiments. Statistical analysis was performed using one-way ANOVA followed by Tukey’s post hoc test. **p ≤ 0.01, ****p ≤ 0.0001

To further assess whether gingipain-dependent BBB disruption results from a loss of TJ integrity, we visualized Claudin-5 and Zo-1 expression in the cerebral vasculature of *Tg(fli1:EGFP)*^*y1*^ zebrafish larvae following larvae injection with individual gingipains. Representative confocal images show that in A-buffer-injected (control) and RgpA-injected larvae, Claudin-5 strongly colocalized with EGFP-labeled vessels (Fig. 7A), while in larvae injected with RgpB or Kgp, Claudin-5 immunolabeling was restricted to short vessel segments or was completely absent in the brain vasculature. Higher-magnification *in vivo* imaging confirmed clear gaps in Claudin-5 labelling along cerebral vessels in RgpB- and Kgp-injected larvae, with Kgp causing the most pronounced alterations (Fig. 7B).

Quantitative analysis demonstrated that mean Claudin-5 fluorescence intensity within cerebral vessels dropped significantly after treatment with RgpB and Kgp compared to control and RgpA-injected larvae (Fig. 7C). Mean EGFP fluorescence intensity in the cerebral vasculature was also markedly reduced in these groups.

**Fig. 8:**
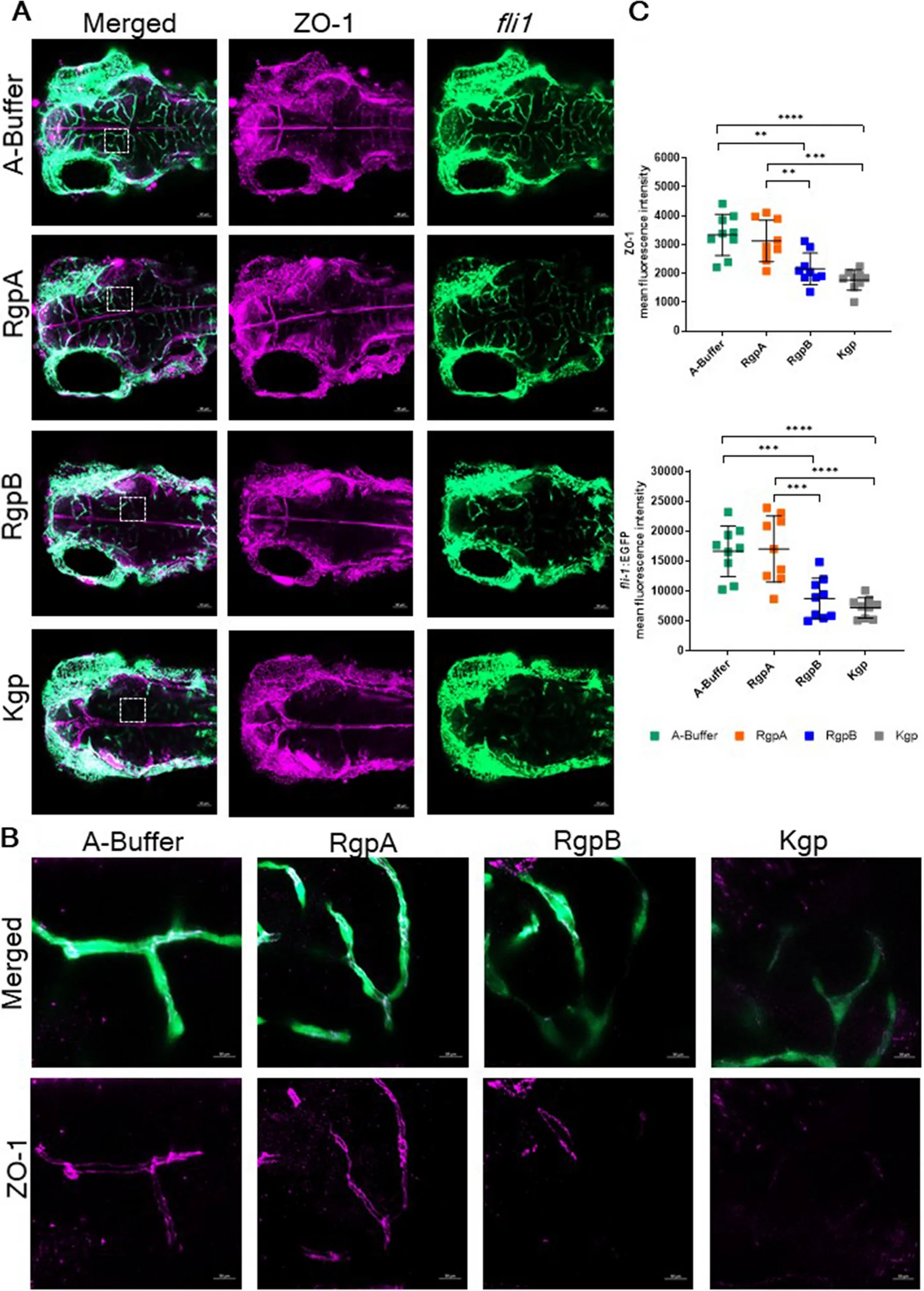
Zo-1 protein expression at the BBB is decreased after systemic injection of RgpB and Kgp, but not RgpA. Transgenic *Tg(fli1:EGFP)*.^*y1*^ zebrafish larvae with fluorescently labeled vasculature (green) were systemically injected with RgpA, RgpB, Kgp, or A-buffer at 3 days post fertilization (dpf). Changes in Zo-1 protein expression was evaluated by IHC staining of fixed larval heads collected at 24 hpi. **A** Representative confocal images show Zo-1 immunoexpression (magenta) within cerebral vasculature in RgpA-, RgpB-, Kgp-, or A-buffer-injected larvae. **B** Magnified representative regions indicated by white dashed boxes in (**A**). **C** Quantification of Zo-1 and EGFP mean fluorescence intensity within cerebral vessels. Data were obtained from at least two independent experiments (*n* = 9–12 larvae per group). Each dot represents one larva. Statistical analysis was performed using one-way ANOVA followed by Tukey’s post hoc test. **p ≤ 0.01, ***p ≤ 0.001, ****p ≤ 0.0001

We next assessed the effect of systemic gingipain injection on Zo-1 protein levels and found that Zo-1 continuously lined cerebral vessels in control and RgpA-injected larvae, but appeared fragmented in RgpB-treated larvae and was nearly undetectable in those treated with Kgp (Fig. 8A and B). Quantification confirmed significantly lower Zo-1 mean fluorescence intensity in RgpB- and Kgp-treated larvae compared to control and RgpA-injected ones, with Kgp showing the strongest effect (Fig. 8C). Similarly, EGFP signal in cerebral vessels was significantly reduced in RgpB- and Kgp-treated larvae, indicating vascular degradation.

Western blot analysis revealed reduced levels of Zo-1 in the heads of gingipain-treated larvae, with the most substantial effect induced by Kgp and the smallest by RgpA. Neither lysine-specific nor arginine-specific protease injections reduce Claudin-5 level (Supplementary Fig. 4S).

#### TJ gene expression remains unaffected in gingipain-treated larvae

To further examine whether the observed BBB disruption upon systemic gingipain injection involves transcriptional changes in TJ, we analyzed the expression of *cldn5a*, *cldn5b*, *tjp1a*, and *tjp1b* genes in the larval brain. Consistent with the results observed after *Pg* infection, systemic administration of individual gingipains (RgpA, RgpB, or Kgp) did not significantly affect the expression of any of the examined genes at either 24 or 48 hpi compared to A-buffer-injected control larvae (Supplementary Fig. 5S). These findings suggest that gingipain-dependent BBB impairment is not mediated by transcriptional downregulation of TJ genes but instead results from direct or indirect (via activated host proteases) degradation of TJ proteins.

## Discussion

Our results demonstrate that *P. gingivalis* infection affects BBB integrity by degrading TJ proteins: Claudin-5 and Zo-1, and consequently increases cerebral vessel permeability. Moreover, for the first time we observed that *P. gingivalis* virulence factors—gingipains are involved in this process, either directly or indirectly, as in gingipain-treated larvae we observed disrupted cerebral endothelial barrier, what potentially allows harmful substances to enter the brain parenchyma and promote neuroinflammation. Our study supports previous discoveries linking infections with periodontal pathogens to neurodegeneration and show that the BBB is an early target during systemic infection with *P. gingivalis*.

Epidemiological studies have long indicated a correlation between chronic periodontitis (CP) and AD, though the underlying biological mechanisms remained elusive [28]. Recent investigations have detected *P. gingivalis* in post-mortem brain tissue and cerebrospinal fluid of AD patients, highlighting its potential to colonize the central nervous system (CNS) [13]. Gingipains have been localized near neurons, tau tangles, and β-amyloid plaques, further implicating this pathogen in AD progression [28]. Moreover, several studies suggested that BBB impairment, which may persist for years before clinical symptoms appear, plays a key role in the early stages of cognitive decline and the progression of AD [29]. Our data extend these findings by providing direct *in vivo* evidence that systemic exposure to *P. gingivalis* results in rapid BBB disruption. Using fluorescent tracers of different molecular mass (DAPI, 350 Da, and FITC–dextran, 70 kDa) to detect both subtle and severe BBB impairment, we observed a significant increase in BBB permeability in zebrafish larvae following intravenous injection of the wild-type *P. gingivalis* W83 strain. This effect was not detected in larvae exposed to the gingipain-deficient mutant (*ΔK/R-ab*), indicating that gingipain activity is important for BBB impairment. These observations provide functional support for the hypothesis that, once *P. gingivalis* enters the bloodstream, it can affect the blood–brain barrier in a gingipain-dependent manner.

Notably, the *ΔK/R-ab* mutant did not induce any measurable increase in BBB permeability, despite expressing other *P. gingivalis*-associated virulence factors. As demonstrated in our previous work [30], the *ΔK/R-ab* mutant is rapidly cleared from the zebrafish circulation via macrophage-mediated phagocytosis. Such rapid elimination likely limits its interaction with brain endothelial cells and prevents barrier disruption. While we cannot completely exclude minor contributions from other bacterial virulence factors, our results strongly support gingipains as important drivers of BBB impairment in this model.

To investigate the mechanism underlying increased BBB permeability, we performed immunohistochemical (IHC) and Western blot analyses of TJ components in the larval brains. A pronounced reduction in Claudin-5 and Zo-1 immunoexpression was observed in the brains of larvae infected with wild-type *P. gingivalis*, whereas no changes were detected in the brains of *ΔK/R-ab*-injected group. However, Western blot analysis of whole-head lysates showed a significant decrease only in ZO-1 protein levels, while Claudin-5 remained unchanged. This discrepancy may be explained by the presence of two paralogs of Claudin-5 in zebrafish: Claudin-5a and Claudin-5b, which differ in their expression patterns and functional relevance.

Recent studies have demonstrated that Claudin-5a is the functional ortholog of mammalian Claudin-5 and is essential for the integrity of BBB. Loss of Claudin-5b showed no effect on zebrafish vasculogenesis or BBB function, whereas the knockout of claudin-5a caused a lethal phenotype of severe whole-brain oedema, ventricular dilatation, and cerebral hernia in zebrafish larvae [31]. In our model, the antibody used for immunohistochemistry likely detects both paralogs, resulting in a visible reduction in Claudin-5 signal at the vascular level, while the Western blot may preferentially detect the more abundant or stable isoform, most likely Claudin-5b, which does not undergo degradation upon infection. Furthermore, Claudin-5a is tightly localized at endothelial junctions and may be more susceptible to proteolytic cleavage or redistribution during inflammation, leading to reduced immunoreactivity without a corresponding drop in total protein levels. Claudin-5b, being less functionally involved in BBB sealing, may remain unaffected and dominate the Western blot signal.

These findings underscore the importance of considering paralog-specific expression and localization when interpreting tight junction protein dynamics in zebrafish models. They also highlight the need for isoform-specific antibodies and transcript-level validation to fully resolve the molecular mechanisms underlying BBB disruption.

Our IHC results are consistent with *in vitro* studies showing gingipain-mediated degradation of junctional and adhesion proteins in endothelial cells [15, 18], and extend those findings to a physiologically relevant vertebrate model. Very recent studies of Jiang and coworkers [32] also found that in mice *P. gingivalis*-induced periodontitis enhanced BBB permeability, measured as Evans Blue leakage, and downregulated the expression of Occludin and ZO-1. However this study focused only on the wild type bacteria and did not explain the role of gingipains in this phenomenon.

Under physiological conditions, the vertebrate BBB allows passive diffusion of small molecules, typically below 400 Da [33, 34]. Thus, the minor DAPI leakage observed in control larvae likely reflects normal paracellular transport. This diffusion is tightly regulated by Claudin-5, the most highly expressed member of the claudin family in brain endothelial cells [20]. The rapid and widespread DAPI leakage observed in larvae infected with wild-type *P. gingivalis* suggests an early disruption of Claudin-5, as supported by the marked reduction in Claudin-5 immunoexpression within cerebral vessels. In parallel, we also observed degradation of Zo-1, a scaffolding protein that anchors tight junction complexes to the endothelial cytoskeleton [35]. Since Zo-1 is essential for the structural stability of the BBB, its loss further supports a breakdown of junctional architecture in response to *P. gingivalis*. The reduction in both Claudin-5 and Zo-1 immunoexpression was not local, but affected the entire brain vasculature, indicating widespread protease activity. This finding is further supported by the extensive leakage of high-molecular-weight FITC–dextran (70 kDa) across the larval brain. While low-molecular-weight dextrans, particularly 10 kDa, are commonly used to detect BBB disruption, permeability to a 70 kDa tracer indicates a profound compromise of barrier integrity in our model [36].

These findings are consistent with our previous observations in transgenic zebrafish larvae expressing fluorescently labelled adhesion proteins (PECAM-1 and VE-cadherin) [21]. Upon infection with wild-type *P. gingivalis*, but not the *ΔK/R-ab* mutant, we observed a marked reduction in PECAM-1 or VE-cadherin fluorescence accompanied by increased dextran leakage in the caudal vein, further underscoring the contribution of gingipains to vascular damage. In this study reduction in PECAM-1 or VE-cadherin fluorescence was observed specifically in endothelium proximal to wild type bacteria, but not gingipain-deficient ones, what suggests destructive effects of gingipains. Also study in rodents highlights the role of *P. gingivalis* in increasing vascular permeability by reducing PECAM-1 immunoexpression in mouse lung, liver, kidney and spleen blood vessels, and shows that this effect is gingipain-dependent [37].

Another study by Farrugia et al. [38] compared the effects of outer membrane vesicles (OMVs) derived from wild-type *Pg* W83 and ΔK/R-ab mutant using zebrafish model, but only in relation to disease symptoms. Although, the results supported our previous *in vivo* observations regarding gingipains involvement in endothelial dysfunction, the effects of *Pg* W83 and ΔK/R-ab-derived OMVs on endothelial cells were examined exclusively *in vitro* using human microvascular endothelial cells (HMEC-1). This study demonstrated that OMVs from wild-type *Pg *W83 significantly reduced levels of the intercellular adhesion molecule PECAM-1, suggesting possible proteolytic cleavage of endothelial cell–cell adhesins by gingipains. However, it has to be mentioned that OMVs contain all gingipains but also other *Pg* virulence factors such as LPS and fimbriae, and therefore do not allow for the identification of the precise mechanism of *Pg*/gingipain actions. In turn, study of Adewoyin and colleagues (2024) showed that in adult zebrafish i.p. injection of *Pg* OMVs induced histopathological changes in the brain and increased neuroinflammation, characterized by elevated expression of nitric oxide (NO), IL-1β, and IL-6, in adult zebrafish however this study did not investigate BBB permeability and TJ expression and did not focused on gingipains. Recently, Qiu et al. [39] also demonstrated, using a mouse model of bacteremia, that Pg-derived OMVs, but not *Pg* bacteria alone injected separately via the tail vein, were able to disrupt TJs of microvascular endothelial cells in the hippocampus. In this study, a decreased level of occludin was detected in the brains of mice injected with *Pg* OMVs, but not in mice injected with Pg alone, as shown by Western blot and immunofluorescence staining. However, these results are not entirely consistent with our findings as we observed a clear increase in cerebral vessel permeability accompanied by a reduction in TJ protein level following systemic injection of *Pg* bacteria. One possible explanation for this discrepancy is that Qiu and co-workers used a different *Pg* strain—ATCC 33277, which is widely recognized as less virulent and highly fimbriated strain responsible for more localized infection. In contrast, we used the highly virulent W83 strain [40] [Naito et al., 2008]. Our unpublished data confirmed that, in contrast to *Pg* W83 infection, systemic infection with *Pg* ATCC 33277 had no effect on cerebral vasculature morphology.

Our analysis of TJ gene expression revealed no differences between infected and control larvae. Transcript levels of *cld5a*, *cld5b*, *tjp1a*, and *tjp1b* remained unchanged following systemic infection with either wild-type or mutant *P. gingivalis*. This indicates that TJ loss is driven by post-translational degradation rather than transcriptional suppression. Although TJ proteins begin forming functional barriers at 3 dpf in zebrafish, their expression undergoes considerable change during larval development as the BBB matures [20, 34]. Despite this developmental variability, we did not observe any infection-related changes in gene expression.

These findings are in line with our previous *in vitro* study in human primary endothelial cells (HCAEC and HDMEC), where exposure to wild-type *P. gingivalis* did not alter PECAM-1 or VE-cadherin mRNA levels [21]. Although some studies [e.g. [41]] suggest that TJ gene expression may increase in response to vascular barrier injury as a compensatory mechanism, such upregulation was not observed in our in vivo system.

Finally, systemic infection with wild-type *P. gingivalis* resulted in a marked reduction in both the number and diameter of cerebral vessels. Dextran-based microangiography revealed that several vessels appeared completely occluded. Additionally, fluorescence corresponding to the endothelial-specific marker *kdrl* was significantly reduced in larvae exposed to wild-type *P. gingivalis*. These findings suggest that *P. gingivalis* not only disrupts TJ integrity but also affects vascular structure.

Previous studies have demonstrated that gingipains can invade endothelial layers and trigger apoptosis through multiple molecular pathways [18]. Notably, [42] showed that TLR–NF-κB signaling plays a central role in maintaining vascular endothelial homeostasis, and that its dysregulation by *P. gingivalis* contributes to endothelial dysfunction. Since endothelial cell loss compromises vessel lumen stability, such cell death may cause the observed narrowing or collapse of cerebral vessels [43]. In our model, these effects were absent in larvae injected with the *ΔK/R-ab* mutant, highlighting the role of gingipains in endothelial damage. This finding is further supported by other studies identifying gingipains as key mediators of endothelial apoptosis [44].

Given the important role of gingipains in BBB disruption demonstrated in our model, we further investigated the individual contributions each of the three gingipains: RgpA, RgpB, and Kgp, to cerebral vessel pathology. All three enzymes are cysteine proteases with potent proteolytic activity, however they differ in their catalytic specificity and domain structure. Kgp is a lysine-specific enzyme, while RgpA and RgpB are arginine-specific enzymes. RgpA and Kgp both contain hemagglutinin/adhesin (HA) domains involved in adhesion to the to host cells and in the bacterial coaggregation, whereas RgpB consists only of the catalytic domain [15, 16, 45]. In our study, the strongest effect was observed in Kgp-injected larvae, a lower effect in RgpB-injected larvae, and almost no impact was found in RgpA-treated larvae. Although both Claudin-5 and Zo-1 contain several lysine and arginine residues that could serve as cleavage sites, the observed differences in their susceptibility may reflect selective interactions between gingipains and the endothelial cell surface. As previously proposed by Katz et. al. [46] in the context of epithelial cells, protease binding may depend on specific surface signals that influence substrate recognition accessibility. However, it has to be mentioned that both Kgp and RgpA possess HA domains, which may suggest that HA-mediated adhesion is not the key determinant of TJ degradation. Instead, it indicates that the catalytic domain and its substrate specificity play the central role in this process. The stronger activity of purified RgpB compared to RgpA can be explained by the simpler chemical structure of RgpB, which contains only the catalytic domain. This domain remains highly available to substrates, whereas the large HA domains of RgpA may to some extent mask the active site and reduce hydrolytic efficiency of the enzyme. As previously shown in an *in vitro* study with keratinocytes, RgpB rapidly degraded cell surface proteins, while RgpA exhibited much slower proteolysis [47]. Consequently, despite having almost identical catalytic domains and the same proteolytic specificity, RgpB is more effective under *in vitro* conditions. Our data also suggest that Kgp, which cleaves after lysine residues, shows the highest efficiency against TJ proteins such as ZO-1 and claudin-5. Consistent with our findings, a previous study by Katz et al., [46] identified Kgp as the principal gingipain responsible for degrading adherence junction proteins, particularly E-cadherin, underscoring its central role in the disruption of intercellular organization. Further support for the selective role of Kgp in TJ degradation was provided by Nonaka et al. [48], who demonstrated that recombinant human GST–occludin was extensively degraded upon exposure to *P. gingivalis* culture supernatant. This effect was not affected by KYT-1, an Rgp-specific inhibitor, but was almost completely eliminated by KYT-36, a selective Kgp inhibitor, implicating Kgp as the main protease responsible for occludin cleavage.

In contrast, the same study reported that both Rgp and Kgp contributed comparably to the degradation of recombinant human Zo-1, which aligns with our observations. In our zebrafish model, exposure to both Kgp and RgpB resulted in a significant reduction of Zo-1 immunoexpression, suggesting that Zo-1 is broadly susceptible to gingipain-dependent proteolysis. The different susceptibility of TJ proteins may result from variations in their structure or tissue localization, which affect how easily gingipains can access and cleave them.

While all three gingipains are capable of degrading host proteins, their impact on endothelium likely extends beyond direct proteolysis. Gingipains are also known to induce chronic pro-inflammatory responses, which can compromise vascular homeostasis [49]. In addition, they have been shown to activate host protease-activated receptor 2 (PAR2) signaling leading to the induction of matrix metalloproteinases (MMPs), which degraded adhesion molecules such as cadherins [18]. This secondary, indirect mechanism may impair junctional structure, further contributing to vascular barrier dysfunction and endothelial damage. However, it needs to be mentioned that in the *in vitro* study on hCMEC/D3 cells a significant reduction in ZO-1 and occludin levels was still observed even after treatment with a PAR2 peptide antagonist and MMP-9 inhibitor. This observation suggests that the direct proteolysis by gingipains represents the predominant mechanism of TJ protein loss within the BBB.

The marked reduction in Claudin-5 and Zo-1 immunoexpression observed following exposure to RgpB, and most prominently to Kgp, was accompanied by increased BBB permeability, as indicated by the leakage of both low- and high-molecular-weight tracers (350 Da DAPI and 70 kDa FITC–dextran) into the brain parenchyma. These results may support the hypothesis that gingipain activity compromises BBB integrity through targeted degradation of TJ proteins, thereby failing the paracellular barrier of cerebral vessels.

Importantly, this effect does not appear to be driven by transcriptional downregulation, as gene expression levels of *cld5a*, *cld5b*, *tjp1a*, and *tjp1b* remained unchanged in larvae exposed to individual gingipains. Taken together with our previous findings comparing wild-type and *ΔK/R-ab* mutant *P. gingivalis* strains, these observations advocate that the loss of Claudin-5 and Zo-1 is primarily the result of direct proteolytic cleavage of TJ proteins, rather than changes in transcription.

In addition, systemic administration of Kgp and RgpB, with RgpB causing less pronounced effects, were associated with visible alterations in cerebral vessel morphology, including reduced vessel diameter, impaired patency, and a noticeable decrease in *kdrl*-driven fluorescence, which may reflect changes in endothelial cell viability or structure. These vascular effects were not observed in larvae exposed to RgpA, suggesting that the ability to induce such structural changes may differ between individual gingipains.

We cannot exclude that RgpA and RgpB may require higher concentrations or prolonged exposure to achieve similar levels of cleavage compared to Kgp. However, this hypothesis could not be evaluated in the current study, as purified gingipains remain enzymatically active for no longer than 24 h under the conditions used [46].

Although the exact mechanisms remain to be fully explained, previous studies have demonstrated that gingipains can induce endothelial cell death through apoptotic pathways [18, 24]. Loss of endothelial cells has been implicated in structural vessel changes, including narrowing and collapse of cerebral vessels. While our model does not directly confirm apoptosis, the observed vascular changes are consistent with endothelial dysfunction and suggest that gingipain-dependent damage possibly extends beyond TJ disruption.

Our data support gingipain-dependent degradation of TJ as a major contributor to BBB impairment, however we are aware that additional mechanisms may also facilitate the interaction of *P. gingivalis* with the cerebrovascular endothelium. These include invasion and transcytosis across endothelial cells, paracellular passage enhanced by inflammatory signaling, or transferring within infected immune cells, a strategy commonly described as the Trojan horse mechanism [50]. Moreover, a recent study demonstrated that *P. gingivalis* can increase BBB permeability by modulating the Mfsd2a/Caveolin-1 (Cav-1)-dependent pathway in brain microvascular endothelial cells (BMECs) [51], suggesting a role for vesicle-mediated transcellular transport in this process. Taken together, these findings indicate that *P. gingivalis*–induced BBB dysfunction likely involves both direct enzymatic degradation and broader alterations in endothelial barrier regulation. These approaches include the application of specific host protease inhibitors, especially those that zymogens are known to be activated by gingipains, e.g. kallikrains, clotting factors, matrix metalloproteases, etc. On the other hand, modulation of NF-κB signaling should dissect the direct proteolytic action of gingipains on BBB proteins from those dependent on activation signaling pathways associated with cerebrovascular integrity.

Although the destructive effect of *Pg*-infection on the vascular endothelial barrier has previously been investigated using zebrafish larvae, these studies did not specifically address the blood–brain barrier (BBB). In this context it cannot be forgotten that the structure and molecular composition of the BBB differ significantly from those of the body vascular and our present findings indicate that, next to the disruption of the systemic vasculature, *P. gingivalis* systemic infection can lead to severe disruption of the BBB through gingipain-dependent degradation of TJ proteins. This study establishes a valuable *in vivo* model for investigating oral pathogen and BBB interactions and providing new insights into early cerebrovascular damage that may link peripheral *P. gingivalis* infection to neurodegenerative pathology. As loss of BBB integrity has recently emerged as an early biomarker of cognitive dysfunction and neurodegeneration, our model may support the discovery of novel therapeutic approaches aimed at preserving cerebrovascular integrity and limiting infection*-*associated neurodegenerative processes.

## Supplementary Information

Supplementary Material 1.

Supplementary Material 2.

Supplementary Material 3.

Supplementary Material 4.

Supplementary Material 5.

## Data Availability

The dataset(s) supporting the conclusions of this article is(are) included within the article (and its additional file(s)).
