# Supplementary figures and images for: Porphromonas gingivalis infection induces gingipain-dependent changes in the brain vasculature of zebrafish larvae
Source: Cell Commun Signal. 2025 Nov 27;23:552. doi: 10.1186/s12964-025-02557-6 (PMC12751225; doi:10.1186/s12964-025-02557-6)

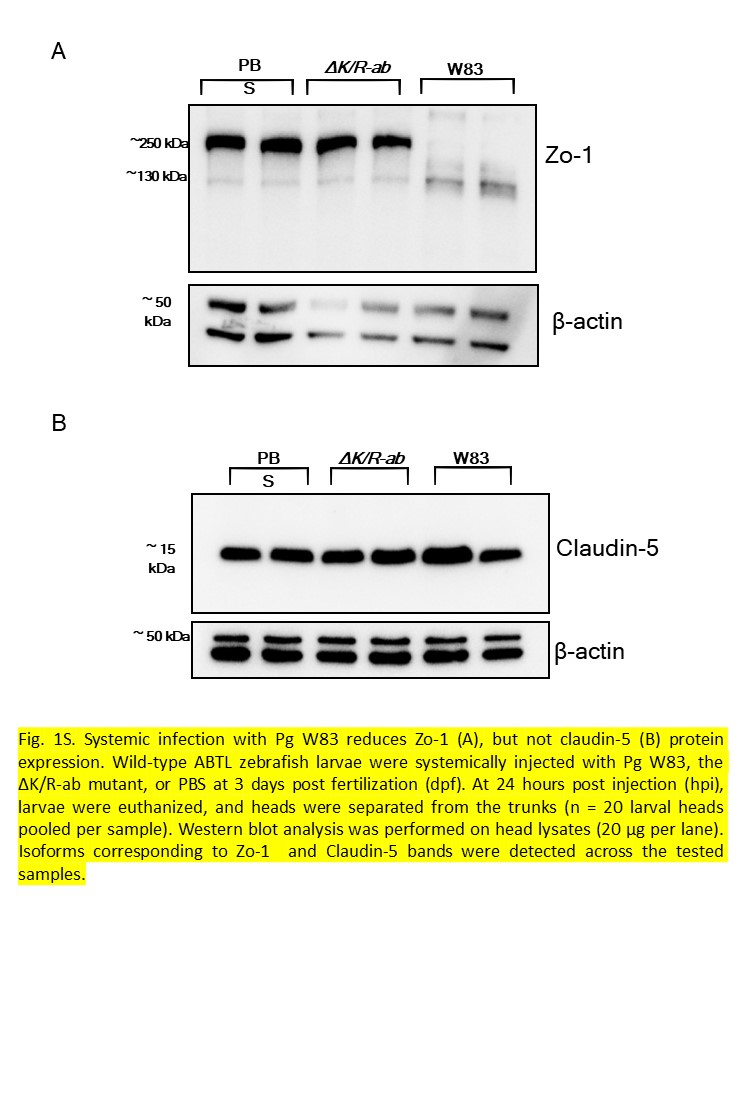

Supplement: Supplementary file 1 — Supplementary Material 1. [file 12964_2025_2557_MOESM1_ESM.jpg]

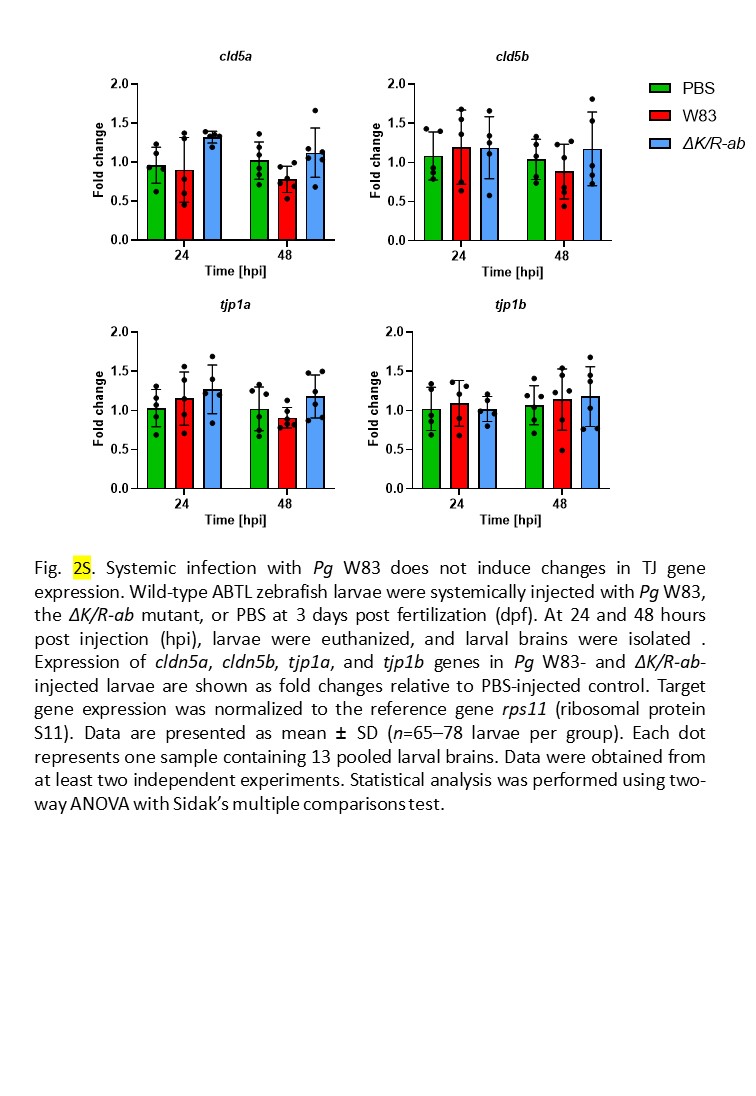

Supplement: Supplementary file 2 — Supplementary Material 2. [file 12964_2025_2557_MOESM2_ESM.jpg]

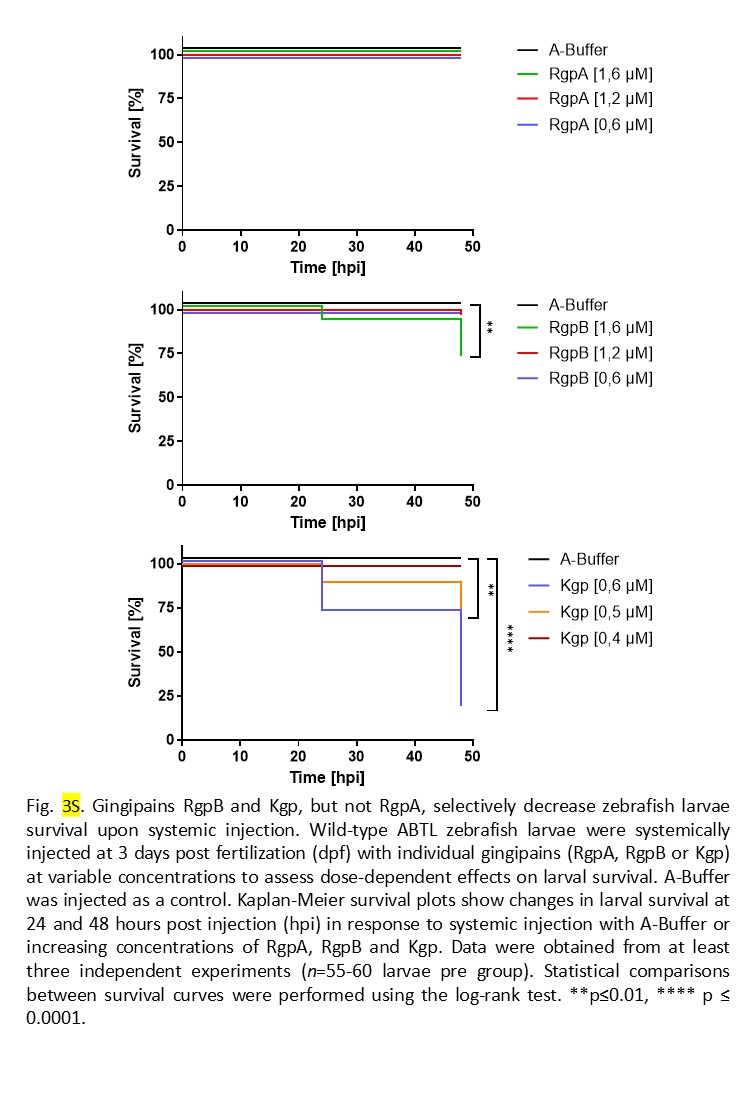

Supplement: Supplementary file 3 — Supplementary Material 3. [file 12964_2025_2557_MOESM3_ESM.jpg]

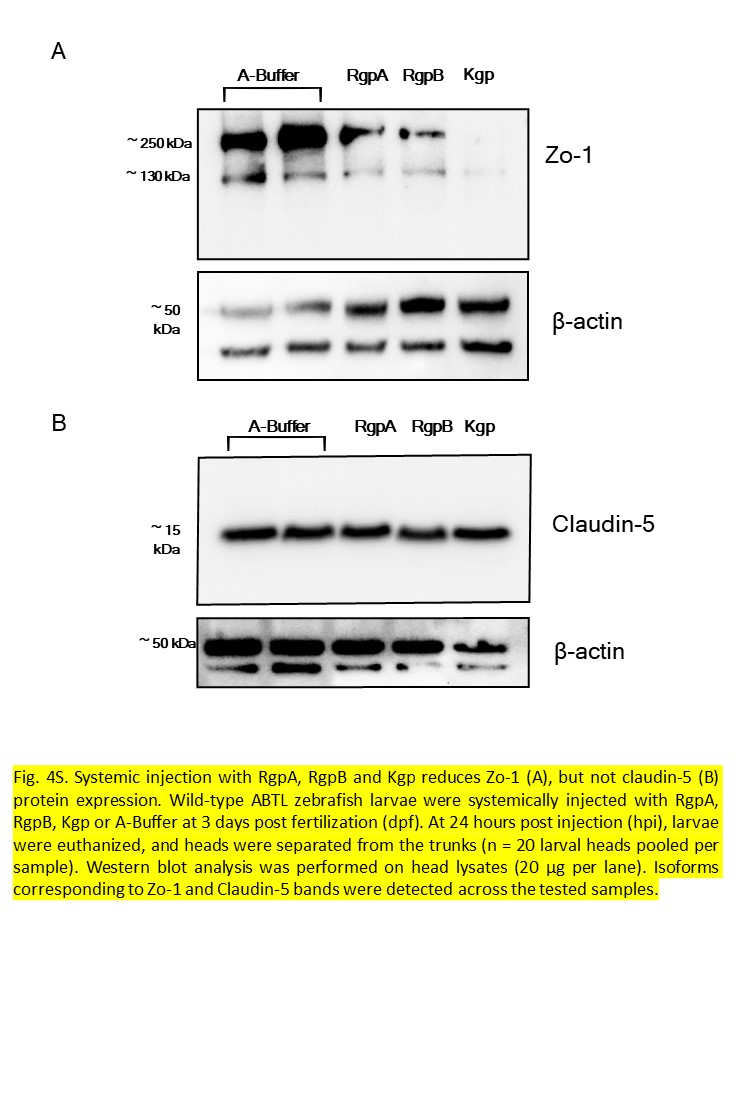

Supplement: Supplementary file 4 — Supplementary Material 4. [file 12964_2025_2557_MOESM4_ESM.jpg]

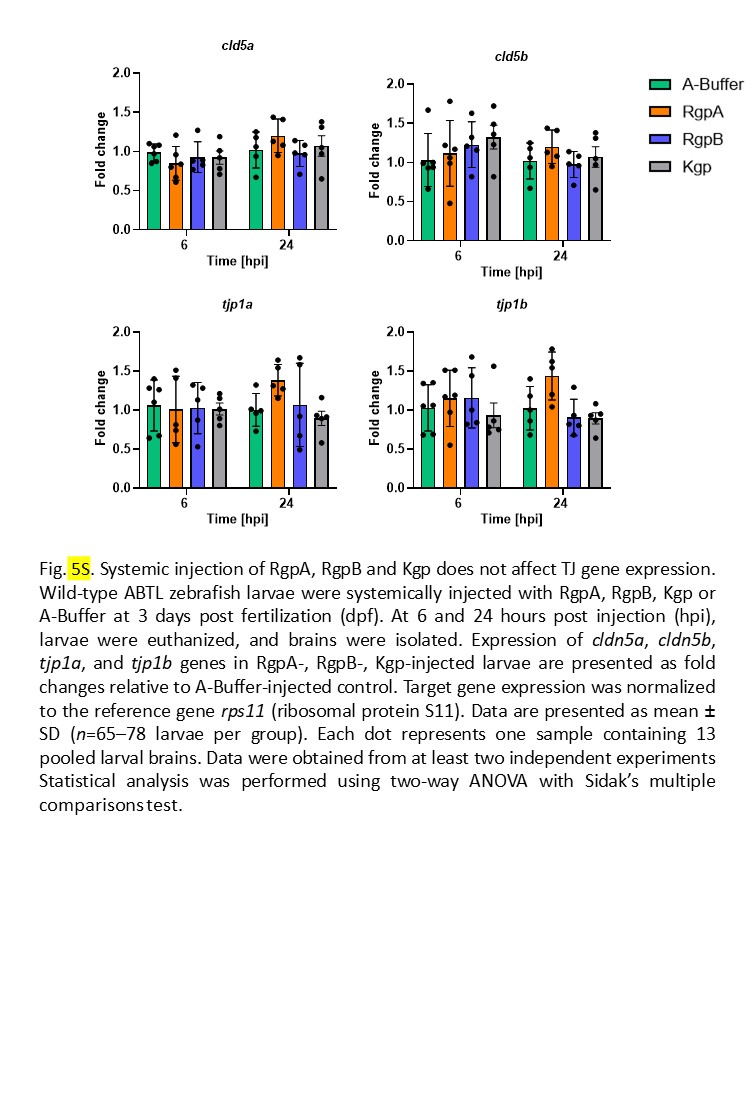

Supplement: Supplementary file 5 — Supplementary Material 5. [file 12964_2025_2557_MOESM5_ESM.jpg]
